# Postbiotics: the route from whey fermentation to functional beverages and food packaging materials

**DOI:** 10.3389/fnut.2026.1943948

**Published:** 2026-10-26

**Authors:** Marija Menkinoska, Zhivka Goranova, Darko Andronikov, Aleksandra Angeleska, Radmila Crceva Nikolovska, Elizabeta Dimitrieska Stojkovikj, Igor Esmerov, Risto Uzunov, Ljupco Angelovski, Nishant Kumar, Anka Trajkoska Petkoska

**Affiliations:** 1 Faculty of Technology, Goce Delcev University, Stip, North Macedonia; 2 Faculty of Food Technology, Institute of Food Preservation and Quality, Agricultural Academy, Sofia, Bulgaria; 3 Faculty of Veterinary Medicine - Skopje, Ss. Cyril and Methodius University in Skopje, Skopje, North Macedonia; 4 Amity Food and Agriculture Foundation, Amity University, Noida, India; 5 Faculty of Technology and Technical Sciences – Veles, University St. Kliment Ohridski Bitola, Veles, North Macedonia; 6 Department of Materials Science and Engineering, Korea University, Seoul, Republic of Korea

**Keywords:** fermentation, food packaging, functional food, Postbiotics, sustainability, waste utilization, whey

## Abstract

Fermented whey beverages are increasingly recognized as promising functional foods due to the presence of fermentation-derived bioactive compounds and, where appropriately processed, postbiotic preparations. Historically, whey was treated as a dairy waste stream and an environmental burden. However, it is a nutrient-rich cheese by-product containing lactose, proteins, vitamins, minerals, and other nutrients, and is therefore an excellent fermentation substrate. Consequently, whey can be effectively valorized for the development of value-added products, supporting circular-economy approaches through the sustainable utilization of dairy by-products. During controlled fermentation, selected microorganisms, such as *Lacticaseibacillus*, *Lactiplantibacillus*, *Limosilactobacillus*, *Lactococcus*, and *Bifidobacterium*, metabolize whey constituents and generate extracellular metabolites and bioactive compounds, while microbial biomass accumulates. When a postbiotic preparation is intended, the biomass is subsequently subjected to a defined inactivation step, with optional cell disruption to expose or recover microbial components, yielding a characterized postbiotic preparation. Fermentation-derived bioactive compounds include enzymes, teichoic acids, organic acids, bioactive peptides, bacteriocins, extracellular polysaccharides, and other substances, which enhance the functional benefits of fermented whey beverages. Although their properties are similar to those of probiotics, postbiotics exhibit improved stability, safety, and efficiency, making them more convenient for food applications. Evidence indicates that postbiotics derived from fermented whey exhibit antioxidant, antimicrobial, and anti-inflammatory properties, as well as the ability to modulate gut health. This review provides a cohesive overview of the formation, composition, and potential biological effects of postbiotic preparations generated from whey through controlled fermentation and subsequent inactivation and processing. It also examines the potential applications of whey-derived postbiotic preparations and fermentation-derived bioactive compounds in active food packaging systems. By summarizing the current knowledge and highlighting research gaps, this review emphasizes the potential value of fermented whey as a postbiotic-rich sustainable resource for the development of novel functional foods and active food packaging systems.

## 1 Introduction

Whey is one of the major by-products generated during cheese production, considered as a significant environmental issue because of its high organic load and disposal difficulties (1, 2). In general, large quantities of whey are produced by the dairy industry worldwide, and improper disposal may contribute to environmental pollution (3). Whey contains various nutritional and bioactive components such as lactose, high-quality proteins, vitamins, and minerals, making it a suitable raw material for the development of value-added functional products (4–7).

In recent years, whey fermentation has attracted growing interest, particularly to produce fermented beverages with potential health-promoting properties (2, 5, 6, 8). Fermentation with lactic acid bacteria can improve the sensory and nutritional characteristics of whey beverages and leads to the production of several bioactive compounds, including fermentation-derived bioactive compounds and metabolites (7, 9). In addition, the conversion of whey into functional ingredients and bioactive metabolites may contribute to a more sustainable use of dairy by-products and support circular economy strategies (10, 11).

Along with the growing interest in fermented functional beverages, increasing attention has also been directed toward postbiotics as potential alternatives to probiotics.

Probiotics and postbiotics are essential in the rapidly advancing field of functional foods and microbiome-focused health approaches, reflecting an increasing understanding of the complex relationship between diet, gut microbiota, and overall health (9, 12). More particularly, probiotics rely on the activity of live microorganisms, such as various strains of lactic acid bacteria (LAB), whereas postbiotics provide bioactive compounds without the presence of live cells. Probiotics are conventionally defined as “live microorganisms which, when administered in adequate amounts, confer a health benefit on the host” (13). Typically, microorganisms commonly used as probiotics include genera, such as *Bifidobacterium*, *Lacticaseibacillus*, *Lactiplantibacillus*, *Limosilactobacillus*, *Lactococcus*, *Leuconostoc*, *Pediococcus*, *Enterococcus*, and *Streptococcus*, and others (1, 14, 15), along with certain yeasts, for example, *Kluyveromyces marxianus*, *Brettanomyces bruxellensis*, *Brettanomyces claussenii* and *Saccharomyces cerevisiae* (4). LABs in whey are primarily used to fermentation of the lactose in dairy by-product, transforming it into lactic acid, which helps stabilize it and improve the flavor and nutritional value of whey-based beverages (1, 4, 6). Probiotics can produce antimicrobial compounds, such as bacteriocins (9), and organic acids (16), modulate inflammatory responses (17), and aid in nutrient metabolism, including the production of vitamins and SCFAs (1). The positive effects of probiotics are achieved through various mechanisms, such as adjusting the gut microbial balance, surpassing harmful pathogens, enhancing the intestinal barrier, and interacting with the host immune system (9, 12). Their efficacy is highly affected by factors such as LAB strain specificity, methods of production, viability during storage and processing, and persistence in the gastrointestinal tract (12, 18). Despite their well-proven benefits, probiotics have certain limitations, particularly in terms of stability and safety for consumers with compromised immune systems (12, 17).

The recognized challenges of probiotics have increased interest in alternatives, such as postbiotics, a novel concept that can deliver comparable benefits without the use of live cells. This concept encompasses non-viable microbial cells, microbial fractions, or cell lysates containing bioactive compounds or metabolites that mimic probiotics’ properties and mechanisms of action (19, 20). Postbiotic preparations may contain inanimate microorganisms and/or their components, including cell wall structures, peptidoglycan, lipoteichoic acids, surface-layer proteins, extracellular polysaccharides, and other microbial-derived cellular constituents (3, 19, 21, 22). These components may contribute to biological activity through interactions with host cells and modulation of immune, metabolic, or barrier-related functions (2, 7, 23). In addition, microorganisms can produce a wide range of metabolites during growth and fermentation, including organic acids, short-chain fatty acids, bacteriocins, γ-aminobutyric acid, vitamins, and other low-molecular-weight compounds (3, 19, 21, 22).

On the other hand, postbiotic preparations do not require viable cells; therefore, they are advantageous in terms of safety, stability, and simplicity for incorporation into food matrices (17, 20). From a technological perspective, postbiotics exhibit improved stability during food processing, including heat treatment, making them particularly favorable for use in processed foods. Additionally, the possibility of determining their chemical composition enables potential for more accurate dosing and standardization compared to live probiotic cells (15, 19, 24). In the recent decade, research has increasingly focused on understanding the source, formation, composition, and bioactivity of postbiotic preparations and fermentation-derived bioactive compounds, and the health benefits gained by their consumption through functional foods (1, 2, 5, 8, 14, 20).

In addition, their thermal and storage stability makes them suitable for incorporation into food-packaging materials, thereby contributing to the preservation of food quality and safety (1, 7, 10–12). The incorporation of postbiotic preparations into food packaging materials represents a promising extension of whey valorization beyond their use as functional food ingredients. Whey proteins are suitable film-forming materials because they can generate transparent, flexible, and edible films with notable oxygen and lipid barrier properties, although their relatively high-water sensitivity and limited mechanical strength may require modification or blending with other polymers (25). These characteristics make whey-protein films and coatings attractive carriers for active antimicrobial compounds. The potential of postbiotic preparations, particularly cell-free supernatants (CFS) from lactic acid bacteria (LAB), for application in food packaging materials arises from their composition, which contains combinations of organic acids, bacteriocins, peptides, enzymes and other microbial-derived compounds (26, 27)

Although numerous reviews have separately addressed whey valorization, probiotics, postbiotics, or fermented dairy products, the available evidence remains fragmented, and an integrated perspective linking these topics is still lacking.

The aim of this review is to provide an overview of fermented whey beverages as sources of postbiotic compounds as well as to discuss their biological activities, functional properties, and potential applications in functional foods and food packaging systems.

## 2 Literature search strategy

Although the aim of the manuscript was not to systematically review the articles related to the topic of fermented whey as a source of postbiotic preparations and their further applications, an article search was performed through databases such as Web of Science and Scopus using pre-defined keywords. The strategy involved using a combination of relevant keywords to capture the scope of the topic. Core search terms included "whey," "whey fermentation," "fermented whey," "dairy by-products," "whey valorization," and "whey-based beverage*". These are combined with microbial and bioactive component-related terms such as "postbiotic*," "probiotic*," "lactic acid bacteria," "bioactive peptide*," "short-chain fatty acid*," "exopolysaccharide*," "bacteriocin*," "organic acid*," "γ-aminobutyric acid," "biosurfactant*," and "phenolic compound*." To address functional and application aspects, terms like "functional food*," "functional beverage*," "food packaging," "active packaging," "food preservation," "antimicrobial*," "antioxidant*," "anti-inflammatory," "gut health," "circular economy," and "sustainable valorization" were included. Additional literature was selected by retrieving citations in the selected articles. The included date range in the search was the period 2016–2026, although a few older articles were also included.

For the selection of studies and data included in this review, the following inclusion criteria were applied to ensure relevance, quality, and focus on the topic: (i) studies focusing on whey as a substrate for microbial fermentation, particularly involving lactic acid bacteria, other bacterial genera, and yeasts; (ii) research articles and reviews related to postbiotic preparations, microbial metabolites, whey-derived biopeptides, and cell-free supernatants; (iii) publications addressing the functional applications of fermented whey and whey-derived postbiotics in food products, including functional beverages and active food packaging; (iv) studies evaluating the biological activities of postbiotics, such as antioxidant, antimicrobial, anti-inflammatory, immunomodulatory, and gut health effects; (v) articles published in peer-reviewed journals and written in English; (vi) research encompassing technological aspects, safety, and regulatory considerations relevant to whey-based postbiotic products.

Articles on research limited to nondairy fermentation substrates or unrelated microbial fermentation processes, publications not accessible in full text, non-peer-reviewed sources, and studies primarily addressing clinical studies without mechanistic or compositional analysis of whey-derived fermentation products were excluded from the review.

This narrative review synthesizes the current knowledge without a systematic eligibility screening or formal quality assessment of the included studies. It does not include a PRISMA diagram or follow a formal systematic literature-selection process. Furthermore, the independent review of articles by two or more authors was not conducted. We identified these as limitations to transparency regarding the identification, screening, inclusion, and exclusion of studies, which may affect the reproducibility and comprehensive coverage of the literature.

## 3 Whey as a substrate for fermented functional beverages

### 3.1 Composition and nutritive characteristics of whey

Whey is the primary liquid byproduct generated during cheese or casein production, representing approximately 85–90% of the total volume of processed milk (10). In the terms of circular bioeconomy, whey represents a valuable biological substrate rather than a simple industrial waste, retaining nearly half of the milk's total solids and approximately 55% of its essential nutrients (11, 28–30). The physicochemical properties of this fermentation substrate are fundamentally determined by the method of milk coagulation, leading to its classification into sweet whey, produced through enzymatic coagulation by rennet at a pH of approximately 6.0–7.0, and acid whey, obtained via lactic fermentation or the addition of organic acids, which lowers the pH to below 5.0 (1, 10, 31, 32).These initial parameters, especially pH and mineral buffering capacity, directly govern downstream bioprocess design and kinetics, as they dictate the selection of robust strains capable of driving efficient microbial metabolism and subsequent cell proliferation (3). The detailed typical composition of whey, its specific protein and nutritional fractions, and their direct relevance to microbial fermentation and bioactive generation are systematically summarized in Table 1.

**Table 1:** Whey composition relevant to fermentation and bioactive generation

Whey component/fraction	Typical composition or examples	Relevance for fermentation	Fermentation-related transformation/bioactive generation	References
Water	~93–95% of liquid whey	Provides the aqueous medium for microbial growth, solute transport and mass transfer	Determines the physicochemical environment and concentration of fermentable and bioactive components	(6, 7, 10, 30)
Total solids	~5–7% of liquid whey	Provides the major pool of carbohydrates, proteins, minerals and minor nutrients available to microorganisms	Influences substrate availability, microbial growth, fermentation kinetics and metabolite formation	(6, 7, 10, 11)
Lactose	~70–75% of dry matter; ~4.5–6% w/v	Principal carbon and energy source for fermentative microorganisms	Hydrolysis to glucose and galactose; subsequent microbial metabolism generates organic acids; transgalactosylation may produce GOS	(6, 10, 11, 30)
Whey proteins	~10–14% of dry matter	Important nitrogen-containing substrate and source of peptides and amino acids required for microbial growth	Proteolysis by microbial proteinases and peptidases generates oligopeptides, free amino acids and whey-derived bioactive peptides	(10, 11, 30, 35)
β-Lactoglobulin	~50–60% of total whey protein	Major protein substrate available for microbial proteolysis	Proteolytic degradation can generate peptide fragments with reported antioxidant and ACE-inhibitory activities	(10, 11, 30, 35)
α-Lactalbumin	~20–25% of total whey protein	Protein and amino-acid source for microbial metabolism	Proteolytic transformation generates peptides and free amino acids; contributes to the nutritional profile of fermented whey	(10, 30, 35)
Bovine serum albumin (BSA)	~8% of total whey protein	Proteinaceous nitrogen source	Can undergo microbial proteolysis and contribute to the pool of fermentation-derived peptides	(10, 30, 35)
Immunoglobulins	~10% of total whey protein	Protein fraction available for microbial/proteolytic transformation	Proteolytic degradation may contribute to peptide formation and the overall functional profile of fermented whey	(7, 10, 30, 35)
Glycomacropeptide	Present mainly in sweet whey	Peptide/protein fraction contributing to the nitrogenous substrate; compositionally distinct from other whey proteins	May undergo microbial transformation and contribute to the nutritional and functional characteristics of fermented whey	(6, 7, 10)
Essential amino acids and BCAAs	Leucine, isoleucine and valine; whey contain all nine essential amino acids	Provide nutrients and metabolic substrates supporting microbial growth and biomass formation	May be incorporated into microbial biomass or participate in strain-dependent metabolic pathways	(6, 10, 34)
Sulfur-containing amino acids	Cysteine and methionine	Provide nitrogen/sulfur and metabolic precursors for microbial metabolism	May participate in microbial metabolic pathways and contribute to glutathione-related nutritional functionality	(6, 10)
Minerals/electrolytes	~7–9% of dry matter; Ca, P, Mg and K are specifically reported	Support enzymatic activity, cell functions, cell-wall integrity and osmotic balance	Influence microbial growth, fermentation performance and the physicochemical properties of the fermented matrix	(6, 10, 30)
B-complex vitamins	Riboflavin (B2), thiamine (B1) and cobalamin (B12)	Provide micronutrients/cofactors relevant to microbial metabolism	Certain LAB strains may synthesize vitamins de novo; these compounds should be regarded as microbially synthesized vitamins/fermentation metabolites rather than postbiotic preparations	(6, 39, 40, 46)
Lipids	~0.05–6% of dry matter	Minor nutrient fraction; contribution depends on whey type and processing history	Potentially subject to strain- and process-dependent transformation, although their role in bioactive generation is less clearly established in the reviewed whey systems	(6, 7, 10)
Sweet vs. acid whey characteristics	Sweet whey: pH ~6.0–7.0; acid whey: pH <5.0	pH and mineral differences strongly influence microbial growth, strain selection and fermentation performance	Acid whey may require acid-tolerant strains or pH adjustment; feedstock differences can affect fermentation kinetics and metabolite production	(10, 28, 30)

In terms of chemical composition, liquid whey consists of approximately 93–95% water and 5–7% total solids, with dry matter predominantly composed of lactose (~70–75%), followed by proteins (~10–14%), minerals (~7–9%), and lipids (~0.05–6%) (6). This composition serves as a highly bioavailable carbon and nitrogen source that directly fuels the microbial growth. The nutritional quality of whey is considered exceptional, with a biological value of approximately 110, which exceeds that of whole eggs, beef, and soy products (5–7, 10, 33). This superior quality is attributed to its complete amino acid profile, which contains all nine essential amino acids required for human physiological functions, as well as its rapid digestibility and absorption into the bloodstream (6). Whey proteins are often referred to as "fast proteins" because of their rapid metabolic availability, which supports human physiological systems and serves as a vital nitrogenous matrix for fermentative strains, enabling rapid cell proliferation and high biomass yields (34).

The protein fraction represents approximately 20% of the total protein content of the original milk and is highly valued for its biological value and functional properties (10). The major proteins include β-lactoglobulin (50–60% of total whey protein), α-lactalbumin (20–25%), bovine serum albumin (~8%), and immunoglobulins (~10%) (6, 30, 31, 35, 36). During fermentation, these specific fractions undergo targeted proteolytic degradation by cell-envelope proteinases and peptidases of starter cultures, yielding a pool of free amino acids and low-molecular-weight peptides that support further microbial growth (35). Individual fractions provide specific precursors that shape the nutritional value of the matrix: α-lactalbumin is rich in tryptophan, a precursor to serotonin and melatonin, whereas β-lactoglobulin serves as a critical transporter of retinol and fatty acids (6). Additionally, sweet whey contains glycomacropeptide (GMP), which uniquely lacks aromatic amino acids such as phenylalanine, making it essential for the nutritional management of phenylketonuria and serving as a selective prebiotic agent during fermentation (29, 33, 37, 38).

Whey is also an abundant source of branched-chain amino acids (BCAAs), specifically leucine, isoleucine, and valine, which act as primary signaling molecules that promote skeletal muscle protein synthesis in preclinical animal models (34). It provides sulfur-containing amino acids, such as cysteine and methionine, which are essential precursors of glutathione synthesis, a significant intracellular antioxidant (6). When subjected to microbial fermentation, the enzymatic machinery of the multiplying biomass hydrolyzes these native whey proteins. This proteolytic pathway (whey proteins → microbial proteolysis → peptide generation) dynamically transforms the matrix, releasing a diverse array of whey-derived bioactive peptides, which must be strictly distinguished from postbiotic preparations that have demonstrated potential antihypertensive and antioxidant properties in vitro (35).

Cheese whey is a rich source of bioavailable electrolytes and minerals, including calcium, phosphorus, magnesium, and potassium (6). Preclinical evidence suggests that consuming cheese whey can support bone mineralization and cardiovascular parameters under specific dietary conditions (34). Crucially, these inorganic ions also function as essential enzymatic cofactors that maintain cell wall integrity and regulate osmotic pressure, thereby directly supporting microbial metabolism and biomass accumulation during fermentation (3). Whey also contains nearly all the water-soluble B-complex vitamins originally present in milk, specifically riboflavin (B2), thiamine (B1), and cobalamin (B12) (10). Riboflavin is often found in higher concentrations in whey than in milk because it can be synthesized de novo by certain lactic acid bacteria (LAB) strains, imparting a characteristic yellowish-green color to the liquid (39). Under a strict postbiotic framework, these vitamins are classified as microbially synthesized vitamins (or nutritionally relevant fermentation metabolites) rather than postbiotic preparations themselves (40).

Despite its exceptional biological value and well-balanced amino acid composition, whey remains a challenging substrate for large-scale utilization because of its compositional complexity and variability. Differences associated with animal species, seasonal factors, feeding practices, and dairy processing technologies can substantially influence the concentrations of proteins, lactose, minerals, and bioactive compounds (10, 28). This inherent feedstock variability directly impacts the reproducibility of microbial fermentations, as fluctuating nutrient ratios can alter starter culture growth kinetics, biomass yields, and the subsequent synthesis of bioactive metabolites (3). Furthermore, the pronounced physicochemical differences between sweet and acidic whey often require distinct processing strategies, increasing technological complexity and production costs (2, 10, 31). Consequently, the broader exploitation of whey in functional applications, specifically in the formulation of standardized fermented beverages, will depend on improved standardization practices, enhanced characterization protocols, and more efficient processing technologies capable of accommodating raw material variability while preserving nutritional functionality and ensuring consistent metabolite profiles (3, 11).

### 3.2 Whey as a fermentation environment

Whey is considered a suitable cultivation medium for many fermentative microorganisms because of its rich lactose content and balanced profile of essential nutrients. When utilized as a fermentation substrate, the composition of the remaining milk solids provides an ideal microenvironment for functional microorganisms, such as LAB and yeasts, which act as a biological "cell factory" to drive active microbial metabolism, generate high-density cellular biomass, and secrete a diverse array of biogenic metabolites (10, 30, 41–43). Historically viewed as an environmental pollutant with a high organic load, the biotechnological valorization of this low-cost, nutrient-dense substrate offers a sustainable pathway to convert organic waste into valuable compounds, thereby reducing the biochemical oxygen demand (BOD) and chemical oxygen demand (COD) of dairy effluents (11, 29, 41).

Lactose, the predominant carbohydrate in whey (~4.5–6% w/v), serves as the primary energy and carbon source to fuel the initial stages of fermentation (10). Microorganisms utilize β-galactosidase enzymes to hydrolyze this disaccharide into glucose and galactose, which are then channeled into intracellular metabolic pathways for cellular growth, biomass accumulation, and organic acid metabolite generation (11). High lactose concentrations support microbial proliferation and biomass expansion and facilitate the synthesis of galacto-oligosaccharides (GOS) via transgalactosylation, adding significant prebiotic value to the final fermented product (1, 11, 30, 39, 44). The accumulation of these oligosaccharides, alongside organic acids and microbially synthesized vitamins, represents key fermentation metabolites that remain within the matrix (40).

Although whey contains fewer total solids and nitrogenous sources than whole milk, it still supplies the essential amino acids and peptides required for sustaining cell viability and driving biomass synthesis (10). The nitrogen fraction is dynamically transformed during fermentation as the cell envelope proteinases and peptidases of starter cultures hydrolyze native whey proteins into extracellular oligopeptides and whey-derived bioactive peptides, which are subsequently transported into microbial cells to support intracellular metabolism (35). Proteolytic activity is essential for auxotrophic strains that require specific amino acids, as it enables their growth and biomass accumulation within the whey matrix (35, 36, 42, 43).

The mineral and vitamin profiles of whey substrate significantly enhance its effectiveness as a cultivation medium for supporting active microbial metabolism and biomass accumulation in LAB. Essential minerals, such as calcium, phosphorus, magnesium, and potassium, serve as critical enzymatic cofactors that support cell wall integrity and osmotic balance, which are vital for sustaining growing cellular biomass (3). Furthermore, the water-soluble B-complex vitamins naturally present in the medium provide the redox potential necessary for maximizing metabolic efficiency and subsequent synthesis of diverse microbial metabolites (6, 33, 40, 42).

The suitability of whey as a fermentation substrate is highly dependent on precise environmental parameters, particularly pH and temperature, which directly govern microbial kinetics, metabolic pathways, and biomass yields (10). The optimal metabolic activity of many LAB strains is generally observed at temperatures between 37°C and 42°C and pH values ranging from 5.5 to 6.5, conditions comparable to those encountered in certain regions of the human gastrointestinal tract (1, 3, 44, 45). In contrast, acid whey, which is characterized by a low pH (<5.0) and higher mineral concentration, presents a more challenging environment for microbial survival (10). This acidic matrix often requires the use of acid-tolerant strains or pH adjustment before inoculation to prevent the inhibition of active biomass proliferation and metabolite synthesis (4).

Supplementation is frequently employed to overcome inherent nutrient limitations, enhance microbial metabolism, and maximize probiotic biomass yields (10). The addition of nitrogen-rich sources, such as yeast extract, or prebiotics, such as inulin, has been shown to significantly enhance biomass production and bacterial metabolic activity during the whey substrate fermentation (3). Furthermore, the synergistic co-culturing of LAB and yeasts (e.g., *Kluyveromyces marxianus*) can improve fermentation efficiency, as yeasts provide essential growth factors, such as vitamins and amino acids, which stimulate bacterial growth and biomass accumulation while contributing to unique aromatic profiles and a diverse pool of microbial metabolites (4).

Importantly, the active fermentation phase does not represent a process of cell lysis. Instead, active fermentation drives the accumulation of live biomass and secretion of extracellular metabolites (46). Following fermentation, where a postbiotic preparation is intended, the accumulated biomass is subjected to a defined inactivation step, such as thermal pasteurization, ultrasonication, or enzymatic treatment, which induces cell inactivation and disruption (22). Depending on the downstream processing applied, the resulting fractions should be distinguished according to their biological origin: fermentation-derived metabolites and whey-derived bioactive peptides remain separately characterized as such, whereas inactivated microbial biomass or defined microbial cellular components may be further processed and standardized as a postbiotic preparation when the resulting material meets the relevant criteria (46). These types of fermentation-derived metabolites and specific inactivation methods used to shape the postbiotic composition are discussed in detail in Section 4.

While the compositional variability of whey remains an inherent challenge, its implications are particularly important in fermentation processes, where differences in nutrient composition can markedly influence microbial growth, biomass yields, cellular kinetics, and metabolite production, thereby affecting process reproducibility (3). Although whey valorization contributes to environmental sustainability and waste reduction, the economic feasibility of fermentation-based systems remains dependent on minimizing the use of costly nutrient supplements and the need for process adjustment (10). Future developments should focus on designing robust microbial consortia and adaptive bioprocessing strategies capable of maintaining consistent performance across diverse whey matrices to ensure stable metabolite profiles (11). Ultimately, recognizing whey as a valuable biological substrate rather than a waste by-product is essential for advancing the sustainable production of next-generation biotic-enriched functional food ingredients and beverages (11).

### 3.3 Whey-based functional beverages

#### 3.3.1 Terminological distinction between postbiotic preparations and fermentation-derived bioactive compounds

The transformation of whey from a high-organic-load effluent into a strategic base for functional beverages represents a cornerstone of the circular bioeconomy in the dairy industry. This shift moves the industry from a "waste-disposal" mindset to one of "value-added" production, utilizing a biological matrix that serves as an excellent fermentation substrate (10). In the context of beverage design, there is a critical terminological distinction between the various products based on their specific composition and processing: fermented whey beverages, which consist of the basic fermented whey matrix; probiotic whey beverages, which must contain viable, living microorganisms throughout their shelf life; defined postbiotic whey beverages, which contain defined, inanimate postbiotic preparation (i.e., inactivated microbial cells and/or their components) that has been appropriately characterized and standardized; synbiotic beverages, which combine probiotics and prebiotic fibers; and metabolite-enriched beverages, which are selectively fortified with specific microbially synthesized metabolites (40). These product categories are summarized in Table 2 according to microbial viability, the presence or intentional incorporation of inactivated microbial components, fermentation-derived metabolites, and whey-derived bioactive peptides, with a specific indication of whether the product can be classified as containing postbiotic preparation.

**Table 2:** Classification of whey-based fermented and functional beverages according to microbial viability, microbial components and fermentation-derived constituents (19, 164, 236).

Product category	Viable microorganisms	Inactivated microbial components	Microbial metabolites	Whey-derived bioactive peptides	Classified as postbiotic preparation*?
Probiotic whey beverage	Present	May be present	May be present	May be present	No, unless a defined postbiotic* preparation is additionally incorporated
Fermented whey beverage	May be present or absent	May be present	Present	May be present	Not by default, only if a defined postbiotic preparation is incorporated
Defined postbiotic whey beverage	Absent	Present and intentionally incorporated	May be present	May be present	Yes, when the postbiotic preparation meets the relevant definition and evidence requirements
Synbiotic whey beverage	Present	May be present	May be present	May be present	Not by default, only if the synbiotic preparation includes a defined postbiotic preparation
Metabolite-enriched whey beverage	Absent or may be present	Usually absent	Present and intentionally enriched	May be present	No, unless a separate defined postbiotic preparation is included

*Postbiotics and postbiotic preparations are understood here as inactivated microorganisms and/or their components, with or without accompanying metabolites, that confer a health benefit on the host when administered in adequate amounts, supported by appropriate evidence.

These different types of beverages aim to deliver targeted health benefits, such as supporting gastrointestinal function and immunological parameters (46). At the component level, lactic acid, GABA, SCFAs, and microbially synthesized vitamins should be described as microbial or fermentation-derived metabolites (40, 46). Peptides released from whey proteins are referred to as whey-derived bioactive peptides (35, 38). Likewise, whole fermented whey should be described as fermented whey or fermented beverage (47), while a cell-free supernatant should be designated as a cell-free fermented fraction unless it meets the criteria for a postbiotic preparation (46, 48). In contrast, inactivated microbial cells and/or defined microbial cellular components may constitute a postbiotic preparation when appropriately characterized and standardized (46, 47).

#### 3.3.2 Formulations, technological challenges, and applications of functional whey beverages

Fermented whey beverages represent a technologically dynamic segment, that utilizes the nutritional components of whey substrate to support the active metabolism, growth, and biomass formation of lactic acid bacteria (LAB) and yeasts, while generating fermentation-derived metabolites and other bioactive compounds (10). Probiotic strains, such as *Lactobacillus acidophilus* LA-5, *Bifidobacterium animalis* subsp. *lactis* BB-12, and *Lacticaseibacillus rhamnosus*, are frequently employed to drive this bioconversion, yielding fermented bases rich in gamma-aminobutyric acid (GABA) and whey-derived bioactive peptides (43). Preclinical in vitro and animal models suggest that these microbially transformed matrices may support gut barrier integrity and modulate inflammatory cytokines (7). Furthermore, whey protein hydrolysis by the active microbial biomass during fermentation releases hydrophobic amino acids, such as tyrosine and tryptophan, which enhance the chemical radical-scavenging activity and overall antioxidant capacity of the fermented matrix, as determined by chemical assays (35). Depending on the downstream inactivation processing applied, this bioactive pool of metabolites, peptides, and inanimate cell components can be standardized into defined postbiotic preparation or formulated directly as a living base for different types of beverages (22).

To overcome the inherent sensory challenges of the native whey substrate, such as its "watery" mouthfeel and slightly salty or acidic taste, manufacturers often incorporate fruit purees and vegetable juices into the final formulations (1, 6). Popular additions include mango, pineapple, grape, peach, orange, and passion fruit, which effectively mask undesirable odors and flavor defects of the substrate while providing essential vitamins, fibers, and polyphenols (1, 11). In addition to fruits, the inclusion of vegetable components, such as carrots, beetroot, and mint has gained traction for developing nutrient-dense, low-calorie functional beverages (41). Recent innovations have also explored the addition of "superfood" ingredients, such as chia seeds, matcha, and turmeric, to boost the antioxidant profile, enhance overall consumer appeal, and provide plant-derived polyphenols (6). Crucially, a clear scientific distinction must be maintained regarding the source of these functional benefits: the antioxidant and bioactive properties of these plant-enriched drinks represent a combination of plant-derived polyphenol effects, the native whey effect, and the fermentation or interactive metabolite effects, rather than being solely attributed to the fermentation process itself (3).

Different types of whey-based beverages are increasingly used in sports and clinical nutrition because of their high BCAA concentrations, particularly leucine, native to the initial substrate (6). Preclinical evidence suggests that these amino acids may support muscle protein synthesis, making them popular in sports formulations (34). Clinical applications have expanded to include specialized drinks for the elderly to prevent sarcopenia (34) alongside nutritional solutions for patients with metabolic disorders, such as type 2 diabetes or phenylketonuria (10, 38). For example, medical-grade whey beverages formulated with specific whey-derived peptides, microbially synthesized vitamins, or inactivated postbiotic preparations have been studied for the dietary management of malnourished patients and those with compromised digestive function (30).

Alcoholic and carbonated variants further diversify the market, enabling the production of different types of beverages, ranging from whey beer and wine to refreshing and naturally carbonated probiotic tonics. Yeasts, such as *Kluyveromyces marxianus*, are often utilized as active biomass in these processes for their unique ability to thrive and metabolize lactose within the whey substrate, contributing to a complex volatilome of fermentation metabolites and aromatic profiles (4, 35, 49).

To preserve the functional integrity of these heat-sensitive proteins and protect the synthesized bioactive compounds during processing, innovative nonthermal and moderate thermal technologies, such as thermosonication (30, 35, 36, 48, 50), and cold plasma treatment (46, 51–53) are replacing conventional thermal treatments. Crucially, these processing technologies can also be evaluated as targeted microbial inactivation approaches for the production of an inanimate microbial preparation, provided that the resulting material meets the predefined criteria for postbiotic preparation (22, 48).

Despite their vast potential, the production of different types of whey-based functional beverages faces several complex technological hurdles that can affect the final product quality and consumer acceptance. One of the primary challenges is the development of bitter off-flavors during the hydrolysis of the whey substrate, caused by the release of specific hydrophobic whey-derived peptides, typically tri- to nonapeptides (35, 42, 54, 55). Physical and structural instability is also a significant concern, as beverages containing fermented bases or postbiotic preparations often suffer from sedimentation, droplet flocculation near the isoelectric point (pH 3-5) of the proteins, and syneresis during storage, resulting in a poor or "watery" mouthfeel (10, 30, 56, 57). Furthermore, acid whey presents a unique complex substrate matrix, including high mineral and acid loads that can lead to membrane fouling during downstream filtration processing and an unpleasantly salty or sour taste in the finished beverage (1, 10, 25, 28, 29, 44).

Maintaining the viability of probiotic strains throughout the shelf life of the product is another major hurdle, as microorganisms must survive environmental stresses such as low pH, oxygen exposure, and nutrient depletion (1, 10, 35). Additionally, this storage vulnerability creates significant process inconsistencies in probiotic drinks, as live-to-dead cell ratios inevitably fluctuate, altering both therapeutic potency and physical stability over time (46). Additionally, the standardization of raw materials is complicated by significant compositional fluctuations caused by differences in animal species, seasonal changes, and the specific diets of lactating animals (6, 10, 33, 41). This feedstock variability poses a direct technological challenge for maintaining reproducible microbial metabolism and standardized metabolite yields across manufacturing batches (3). Finally, the high capital investment required for advanced technologies, such as ultrafiltration, nanofiltration, and enzymatic debittering, creates a significant barrier to scaling production for many small- and medium-sized dairy enterprises (10).

Modern strategies to mitigate these issues include enzymatic debittering using exopeptidases, nanoencapsulation of sensitive ingredients, and fortification of whey with milk or condensed milk to improve the casein structure and viscosity (10, 42, 54, 55, 58). Furthermore, the use of prebiotic fibers, such as inulin or agave inulin, not only improves the textural properties of the beverage but also supports the viability of probiotic strains throughout the product's shelf life (5, 8, 59). By reinforcing the physical matrix and acting as fermentable co-substrates, prebiotics support biomass retention during fermentation and may influence the production of specific fermentation-derived metabolites, depending on the microorganism, substrate composition, and processing conditions (3).

As global consumer demand for healthy, sustainable, and "clean-label" options continues to grow, whey-based functional foods and beverages are poised to become essential components of modern diet. The integration of advanced biotechnological methods, such as synbiotic formulations and GABA enrichment, further enhances the therapeutic potential of these products (5, 10, 11, 30, 43). Ultimately, the successful valorization of whey into functional beverages not only provides a high-quality nutritional resource but also mitigates the environmental impact of one of the dairy industry's most challenging byproducts, aligning perfectly with the principles of the circular economy (3, 10, 11, 60, 61).

While laboratory-scale innovations have demonstrated considerable potential, the transition of whey-based functional beverages to large-scale industrial production remains constrained by high capital investment requirements and the inherent variability of whey as a raw material. The absence of standardized characterization and quality-control protocols frequently results in inconsistencies in product stability, functionality, and sensory properties during storage. Furthermore, many of the reported health-promoting effects are based predominantly on *in vitro* or preclinical animal investigations, emphasizing the need for well-designed human clinical studies to substantiate these claims (46). From an industrial perspective, regulatory uncertainties surrounding postbiotic products, together with the costs associated with purification, stabilization, and debittering processes, continue to limit broader commercial adoption, particularly among small and medium-sized enterprises.

Ultimately, the extensive technological challenges in delivering viable and stable probiotic biomass within these beverages have shifted industrial interest toward postbiotics. These inactivated preparations bypass cell viability bottlenecks, providing comparable health benefits and technical predictability without the safety and shelf-life constraints of live cells (46). Under this framework, the subsequent sections transition from the living substrate environment to explore the biochemical concept of postbiotics, the specific pathways of their synthesis during whey fermentation by active biomass, and the major classes of inanimate components driving functional wellness in next-generation beverages (48).

## 4 Concept, definition of postbiotics, and terminology

Over time, it became evident that a portion of the observed physiological effects could not be attributed solely to viable probiotic cells because structural components and metabolic products emerged as equally relevant contributors to probiotic activity. These insights have sparked a substantive discussion regarding the need for a distinct term encompassing products derived from microorganisms following their inactivation (19, 62). Prior to the establishment of a unified definition, the literature accommodated a range of designations, including para-probiotics, metabiotics, bacterial lysates, inactivated probiotics, and heat-treated probiotics. In the past, the concurrent use of multiple terms for analogous products has generated considerable terminological inconsistency and impeded meaningful comparison of findings across studies (19, 63).

The year 2021 marked a defining moment in the scientific consensus of this field. The International Scientific Association for Probiotics and Prebiotics (ISAPP) published a consensus document intended to establish a clear and unified definition, proposing that a postbiotic constitutes "a preparation of inanimate microorganisms and/or their components that confers a health benefit on the host" (46). This definition requires a known microbial source, a documented inactivation procedure, and confirmed biological activity of the final preparation. This consensus represents a meaningful advance in definitional clarity; however, it simultaneously raises the evaluative threshold, as preparations lacking robust evidence of host benefit cannot legitimately be classified within this category, regardless of their compositional characteristics. Despite its broad acceptance, some authors have argued that the ISAPP definition may exclude certain purified microbial metabolites that exert biological effects but do not necessarily contain inactivated microbial cells or cell fragments (46). The consensus definition provides a clearer distinction between probiotics, prebiotics, and postbiotics, highlighting its importance for both scientific understanding and regulatory frameworks.

Current research suggests that postbiotic preparations may contain inanimate microorganisms and/or their components, including cell walls and surface structures, exopolysaccharides, and other microbial-derived components. Fermentation may also generate bioactive metabolites, such as organic acids, enzymes, and peptides, which can contribute to the biological activity of fermented products or postbiotic preparations when appropriately demonstrated (19, 46). The interest in these preparations is partly grounded in their greater stability during technological processing and storage, as their activity is independent of cellular viability. Available data reveal substantial heterogeneity among postbiotic preparations, and the microbial species, cultivation conditions, growth medium, inactivation method, and final product composition all exert an influence on the observed biological effects (40, 46). Findings established for one preparation cannot be extrapolated to others produced from different microorganisms or under different technological conditions, a limitation that must be explicitly acknowledged when interpreting the existing evidence base. This constraint represents one of the most pressing methodological challenges in the field and underscores the necessity for preparation-specific characterization as the foundation of both scientific evaluation and regulatory assessment of postbiotics as a distinct category of functional products. Understanding the conceptual framework of postbiotics is essential before examining their composition, mechanisms of action, and technological production strategies. Against this background, the following sections examine the generation of fermentation-derived bioactive components during whey fermentation, with particular emphasis on microbial metabolites and other components that may contribute to the biological activity of fermented whey and postbiotic preparations.

## 5 From whey fermentation to defined postbiotic preparations

### 5.1 Microorganisms in whey fermentation for postbiotic preparations

Fermentation is a natural and eco-friendly process that results in products with a broad range of flavors and enhanced nutritional benefits. It aligns with consumer preferences and expectations, steadily gaining attention and popularity.

LAB are one of the most important groups of microorganisms used in food biotechnology, particularly in dairy fermentation. During whey fermentation, LAB reduce the pH of the medium, improve microbiological safety, and produce a broad spectrum of bioactive compounds with functional and health-promoting properties (3, 19, 21, 22, 30, 31). Current taxonomically accepted bacterial names are used throughout the manuscript, while former names are retained only in the titles of cited sources.

Lactic acid bacteria (LAB) are gram-positive, catalase-negative, non-spore-forming microorganisms that primarily produce lactic acid through carbohydrate metabolism. Important industrial genera include *Lacticaseibacillus*, *Limosilactobacillus*, *Lactiplantibacillus*, *Lactococcus*, *Leuconostoc*, *Pediococcus*, *Enterococcus*, *Streptococcus*, and *Bifidobacterium* (1, 3, 14, 30, 39, 64, 65). These microorganisms are generally recognized as safe (GRAS) and have a long history of use in dairy fermentation (65, 66). Yeast strains *Kluyveromyces lactis* (5, 67) and *Saccharomyces cerevisiae* (14) are also used as fermentation cultures for postbiotic production from whey. Based on their fermentation route, LABs are classified into two main groups: homofermentative, which predominantly convert sugars into lactic acid, and heterofermentative, which additionally produce acetic acid, ethanol, carbon dioxide, and aroma compounds (30, 66). This metabolic diversity contributes to the preservation and microbial safety of fermented whey products, as well as their sensory and functional characteristics (4, 66).

The production of postbiotic preparations by LAB is strongly influenced by fermentation conditions, such as temperature, pH, oxygen availability, incubation time, inoculum size, and nutrient supplementation (4, 58). Most LAB used in whey fermentation grow optimally between 30 and 42°C and under mildly acidic conditions (3, 51, 64, 67–71). Furthermore, mixed-culture fermentations involving combinations of LAB strains (30, 35, 51, 72), or combining them with yeasts (73, 74) may outperform single-strain fermentations, thus enhance metabolite diversity and improve postbiotic yield through synergistic interactions. Furthermore, it reduces allergenic proteins and produces bioactive peptides with antioxidant and antihypertensive properties (35, 75).

Figure 1 illustrates an integrated platform for the valorization of whey through controlled fermentation and downstream processing into bioactive ingredients and functional products. The process begins with the selection of an appropriate whey type, including sweet whey, acid whey, lactose whey, and permeate or deproteinized whey. Because these substrates differ in lactose, protein, mineral, and nitrogen-containing compound composition, their characteristics can influence microbial growth, metabolic activity, and the profile of fermentation-derived compounds. Pretreatment steps, such as clarification, thermal treatment, concentration, and pH or mineral adjustment can subsequently be applied to improve substrate stability and optimize conditions for microbial fermentation. This integrated approach leverages precision strain selection, data-driven process control, sustainability, circular economy principles, and regulatory compliance to advance the development of innovative health-promoting whey-derived products.

**Figure 1:**
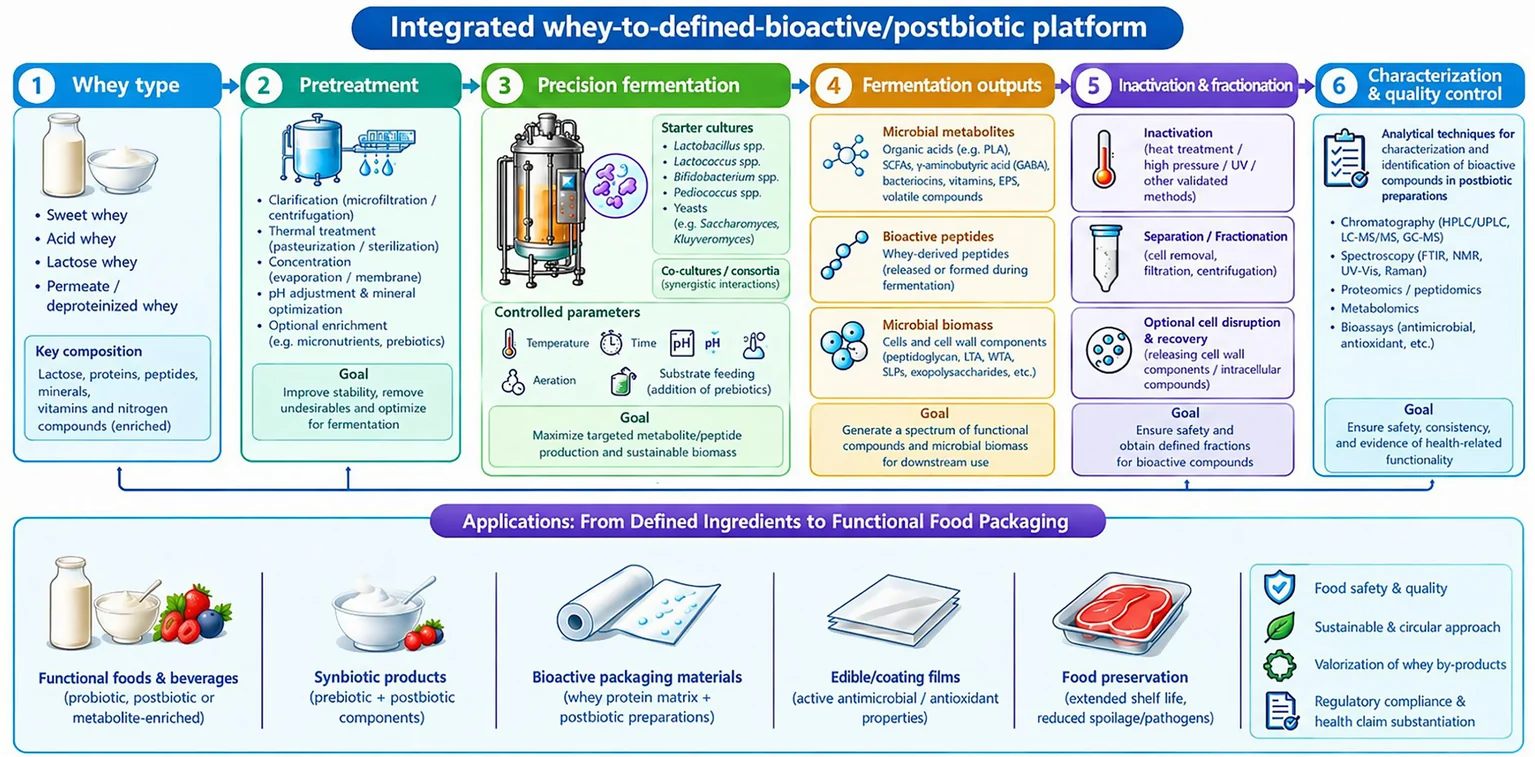
Integrated mechanistic platform for conversion of whey into defined postbiotic preparations and fermentation-derived bioactive ingredients [Created with ChatGPT (OpenAI, GPT-5.5) and edited by the authors]. The schematic figure does not reproduce copyrighted graphical elements. Whey composition determines the available carbon, nitrogen and mineral substrates (6, 7, 10, 11, 30, 35). Pretreatment modifies substrate accessibility, removes inhibitory or undesirable components and adjusts physicochemical conditions for microbial growth (3, 5, 9, 16, 35, 64, 69–71, 76, 83). During controlled fermentation, selected microorganisms convert lactose and other accessible substrates through strain-specific metabolic pathways, generating organic acids, amino-acid-derived metabolites, exopolysaccharides, bacteriocins and other fermentation products, while proteolysis can release whey-protein-derived peptides (1, 3, 14, 15, 30, 39, 43, 65, 67). The resulting fermentation broth therefore contains a heterogeneous mixture of microbial metabolites (1, 3, 30, 42, 93–95), bioactive peptides (36, 38, 54, 145, 146), and microbial biomass (22, 85–88). Downstream inactivation can include processes, such as heat treatment (31, 64, 69, 82), separation/fractionation (3, 16, 19, 35, 70, 83), and optional cell disruption and recovery (18, 21, 31, 81). Characterization of postbiotic preparations involves use of chromatography (3, 5, 70, 88, 115, 117, 136, 149, 150), spectroscopy (3, 5, 7, 68, 87, 88, 117, 127, 131, 132), proteomics/peptidomics (36, 64, 78, 151), metabolomics (104, 127, 152), and bioassays (36, 64, 151). Depending on composition and demonstrated health benefit, the resulting preparations may be developed as fermented foods, defined bioactive ingredients or postbiotic preparations, and may subsequently be incorporated into functional foods, beverages, edible films, coatings or active packaging.

### 5.2 Production of postbiotic preparations

The formation of postbiotic compounds during whey fermentation is a multifaceted process governed by microbial metabolism, substrate composition, and processing conditions. Microbial metabolic and enzymatic processes can transform lactose, proteins, and other whey constituents into a range of potentially bioactive compounds (42, 60). Therefore, the efficiency and quality of bioactive compound production are highly dependent on the optimization of fermentation parameters and downstream processing techniques. In particular, the selection of appropriate microbial strains and gentle yet effective cell inactivation methods are crucial for preserving the bioactivity of target compounds (3, 5, 64, 67). Depending on the microorganisms employed and the fermentation conditions, these processes may generate microbial metabolites, whey-derived bioactive peptides, extracellular polysaccharides, bacteriocins, and microbial cell-associated components. The composition and relative abundance of these compounds are influenced by the whey substrate, microbial strain, fermentation conditions, and downstream processing.

The production process of bioactive compounds from whey begins with controlled fermentation using LAB and other bacterial strains (18, 64, 76, 77), and certain yeast strains (4, 5, 67). Concurrently, secondary metabolic pathways facilitate the production of SCFAs (22, 78) bioactive peptides (BAPs) generated through the enzymatic proteolysis of whey proteins, exopolysaccharides (EPSs) (22, 78) and vitamins (78, 79). The qualitative and quantitative composition of bioactive compounds produced by bacteria and yeasts is strongly influenced by multiple factors, including the microbial strain used, fermentation parameters (pH, temperature, and duration), and substrate characteristics (3, 5, 67) (Figure 1). In addition to conventional liquid fermentation, solid-state fermentation (SSF) is gaining interest as an alternative method for producing BAPs with enhanced yield and purity (31, 80). Both bacterial and yeast strains are frequently used in SSF because of their robust proteolytic systems and acid tolerance, such as co-fermentation of LAB with *Saccharomyces* spp (31, 73).

Following fermentation, LAB inactivation is performed, which is a deliberate step used to kill the microorganisms cultured in whey, aiming to yield a stable preparation of inanimate cells, cell fragments, and metabolic products (30). The selection of inactivation strategies depends on the target metabolites, whether intracellular, extracellular, or a combination of both factors. The recovery of intracellular components, such as teichoic acids, peptidoglycans, and surface layer proteins, requires an initial cell disruption step (18, 31). Cell lysis can be achieved using chemical, physical, or enzymatic methods, each offering specific advantages and limitations (21, 31).

Chemical methods involve extreme pH conditions (e.g., pH ≈ 2), are cost-effective and scalable, but may denature sensitive protein-derived compounds, thereby diminishing their bioactivity (18). Enzymatic lysis, although more time-intensive and costly, employs mild operating conditions and is considered the most effective approach for maintaining the structural integrity and functionality of compounds such as bacteriocins, EPSs, and organic acids (21, 31, 81). However, the lack of knowledge regarding the scalability of the production methods has probably resulted in a scarcity of published research on enzymatic lysis (21). Additional non-thermal technologies for the production fermentation-derived bioactive compounds production that have recently emerged include high-pressure processing, pulsed electric fields, ultrasound, cold plasma, supercritical CO_2_, and irradiation (18, 21, 31).

When the focus is on extracellular metabolites, thermal methods, including sterilization, pasteurization, and ohmic heating, are commonly employed depending on the microbial strain involved in the production of bioactive compounds (31, 64, 69). Heat treatment induces structural alterations in cell membranes, RNA, DNA, ribosomes, and enzymes, resulting in cell death (31). However, elevated temperatures (e.g., 121 °C for 10 min) can compromise proteins and polysaccharides by altering their bioactivity and functional properties (31, 82). In contrast, mild heating (e.g., 55–62 °C for 30 min) effectively inactivates enzymes and disrupts cell membranes while preserving key bioactive characteristics (82). Unlike conventional heat treatments, which require high energy, long durations, and specialized equipment, ohmic heating offers a rapid and more efficient alternative (31, 82). This enhances the functionality of microorganism derivatives by maximizing cell disruption while preserving active metabolite profiles under optimal processing conditions.

Subsequent downstream processing is essential for isolating, purifying, and stabilizing postbiotic-related fractions in the fermentation environment. Ultracentrifugation is widely employed to remove residual microbial biomass because of its efficiency, accessibility, and high recovery rate. Additional purification, harvesting, and stabilization techniques, such as microfiltration, dialysis, chromatographic separation, and spray- and freeze-drying, can be applied to enhance postbiotic quality for incorporation into functional foods (3, 19, 83). Together, these production steps ensure that postbiotic preparations maintain their functional properties, safety, and stability for the intended applications. Table 3 summarizes example studies on bioactive compound production utilizing various bacterial and yeast strains, detailing their fermentation conditions, cell inactivation or removal methods, postbiotic downstream processing, major postbiotic compounds formed, and the analytical methods employed for characterization and quantification.

**Table 3:** Example studies of lactic acid bacteria/yeasts fermentation for bioactive compounds production.

Bacterial/yeast strains	Fermentation conditions	Cell inactivation/removal	Postbiotics harvesting/stabilization	Major bioactive compounds	Characterization and quantification	Reference
*Bifidobacterium lactis* BB12 + linoleic acid in Tween 80	38 ^o^C, 28 h, pH – 4.5, aerobic	Heating at 121 °C for 15 min	Centrifugation, 0.45 μm filtration of CFS	CLA, EPSs, BACs	FTIR, GC, TLC, SDS-PAGE	(64)
*Saccharomyces boulardii* and *Saccharomyces cerevisiae* + trypsin/pepsin	30 ^°^C, 44 h, aerobic	Ultracentrifugation	0.22 μm filtration, freeze drying of CFS	Organic acids, GABA, amino acids, polyphenols, vitamins	UPLC - HRMS	(9)
*Lacticaseibacillus paracasei ABK*	30 ^°^C, 24 h, aerobic	Ultracentrifugation	0.22 μm filtration of CFS	Amino acids, organic acids	Capillary electrophoresis with UV detector	(16)
*Lactobacillus helveticus H9*	37 °C, 24 h, aerobic	Ultracentrifugation	0.22 μm filtration of CFS	Amino acids, organic acids (lactic acid high content)	Capillary electrophoresis with UV detector	(16)
*Lactiplantibacillus plantarum* J26	37 ^°^C, 14 h, aerobic	Pulsed electric field, spray drying	Centrifugation of CFS	Lipids and lipid-like molecules, organic acids and derivatives,	HPLC-HRMS	(83)
*Bifidobacterium longum* BIOCC 1719	37 °C, 24 h aerobic	Centrifugation	No treatment of CFS before UHPLC-MS/MS	B group vitamins, essential amino acids, SCFAs,	UHPLC-MS/MS	(64)
*Lactiplantibacillus plantarum* 299v + 1-2% inulin or chia mucilage	35 ^°^C, 30 h pH-2.4, aerobic	Ultracentrifugation	0.45 μm filtration, freezing of CFS	Lactic acid, SCFAs, phenols	Folin-ciocalteu, HPLC-DAD	(3)
*Lacticaseibacillus casei* Shirota + 1-2% inulin or chia mucilage	35 ^°^C, 30 h pH-2.4, aerobic	Ultracentrifugation	0.45 μm filtration, freezing of CFS	Lactic acid, SCFAs,	Folin-ciocalteu, HPLC-DAD	(3)
*Bifidobacterium animalis* subsp. *lactis* BPL1 + 1-2% inulin or chia mucilage	35 ^°^C, 30 h pH-2.4, aerobic	Ultracentrifugation	0.45 μm filtration, freezing of CFS	Lactic acid, SCFAs,	HPLC-DAD	(3)
*Lactiplantibacillus plantarum* PTCC1745	37 ^°^C, 36-48 h, pH – 4.5, aerobic	Heating 95 ^°^C 5 min, sonication	Centrifugation, 0.45 μm filtration of CFS	Vitamins, antimicrobial proteins, BAPs, biosurfactants, organic acids, bacteriocins, phenolic acids	Particle size dynamic light scattering	(69)
*Lactobacillus acidophilus LA-5* + 0.05% w/v L-cysteine	37 ^°^C, 36 h pH - 4.5, aerobic	Ultracentrifugation	0.45 μm filtration, freeze drying of CFS	Citric, lactic, butyric acid, free amino acids, phenolic compounds	GC-MS, HPLC-DAD Folin-ciocalteu method	(70)
*Lactobacillus acidophilus LA-5* + 3.9% (w/w) lactose	42 ^°^C, 35.5 h, aerobic	Ultracentrifugation	saline solution washing, freeze drying of CFS	EPSs, lactic acid, AMPs, SCFAs, amino acids	SEM	(71)
*Pediococcus pentosaceus* RC007	37 ^°^C, 24-48 h, aerobic	Ultracentrifugation	Acidic treatment of CFS before GC-FID	SCFAs (acetic and lactic acid)	GC-FID	(76)
*Lactobacillus acidophilus* LA3, *Lacticaseibacillus rhamnosus* GG, *Lactiplantibacillus plantarum* 299v, *Lactococcus lactis,* + 0.5% lactose *Kluyveromyces lactis*	37 ^°^C, 24 h, aerobic,	Ultracentrifugation	0.45 μm filtration of CFS	GABA	GABA enzymatic assay	(5)
*Lactobacillus delbrueckii* spp + lactose	30/37 °C for 72 h,	Ultracentrifugation	0.22 μm filtration of CFS	Substances with antioxidant, antimicrobial, and ACE - inhibitory activities	HPLC-RID SE-HPLC	(35)

CLA, conjugated linoleic acid; EPSs, exopolysaccharides; BACs, bacteriocins; FTIR, fourier-transform infrared spectroscopy; GC, gas chromatography, TLC, thin-layer chromatography; SDS-PAGE, sodium dodecyl sulfate–polyacrylamide gel electrophoresis; SCFAs, short-chain fatty acids; CFS, cell free supernatant; HPLC-DAD, high performance liquid chromatography with photo-diode-array detector; HRMS, high resolution mass spectrometry; GABA, γ-amino butyric acid; AMPs, antimicrobial peptides; SEM, scanning electron microscopy; RID, refractive index detector; UHPLC, ultra-high-performance liquid chromatography; FID, flame ionization detector; MS/MS, tandem mass spectrometry; UV, ultraviolet; SE, size exclusion; ACE, angiotensin converting enzyme.

## 6 Molecular composition and characterization of postbiotic preparations

According to the current consensus definition, microbial cellular components, including peptidoglycan, teichoic acids, lipoteichoic acids, surface-layer proteins, and other cell-associated structures, may constitute components of a postbiotic preparation following appropriate microbial inactivation and processing. In contrast, SCFAs, organic acids, bacteriocins, volatile compounds, microbial enzymes, and other fermentation-derived products should be described as microbial metabolites or microbial-derived bioactive compounds rather than postbiotics per se. Likewise, whey-derived bioactive peptides should be distinguished from microbial cellular components (21, 22, 84). Figure 2 illustrates the molecular origins of bioactive substances produced through whey fermentation by lactic acid bacteria and/or yeasts, presenting the diverse array of whey-fermentation bioactives, which hold significant potential for health and functional food applications.

**Figure 2:**
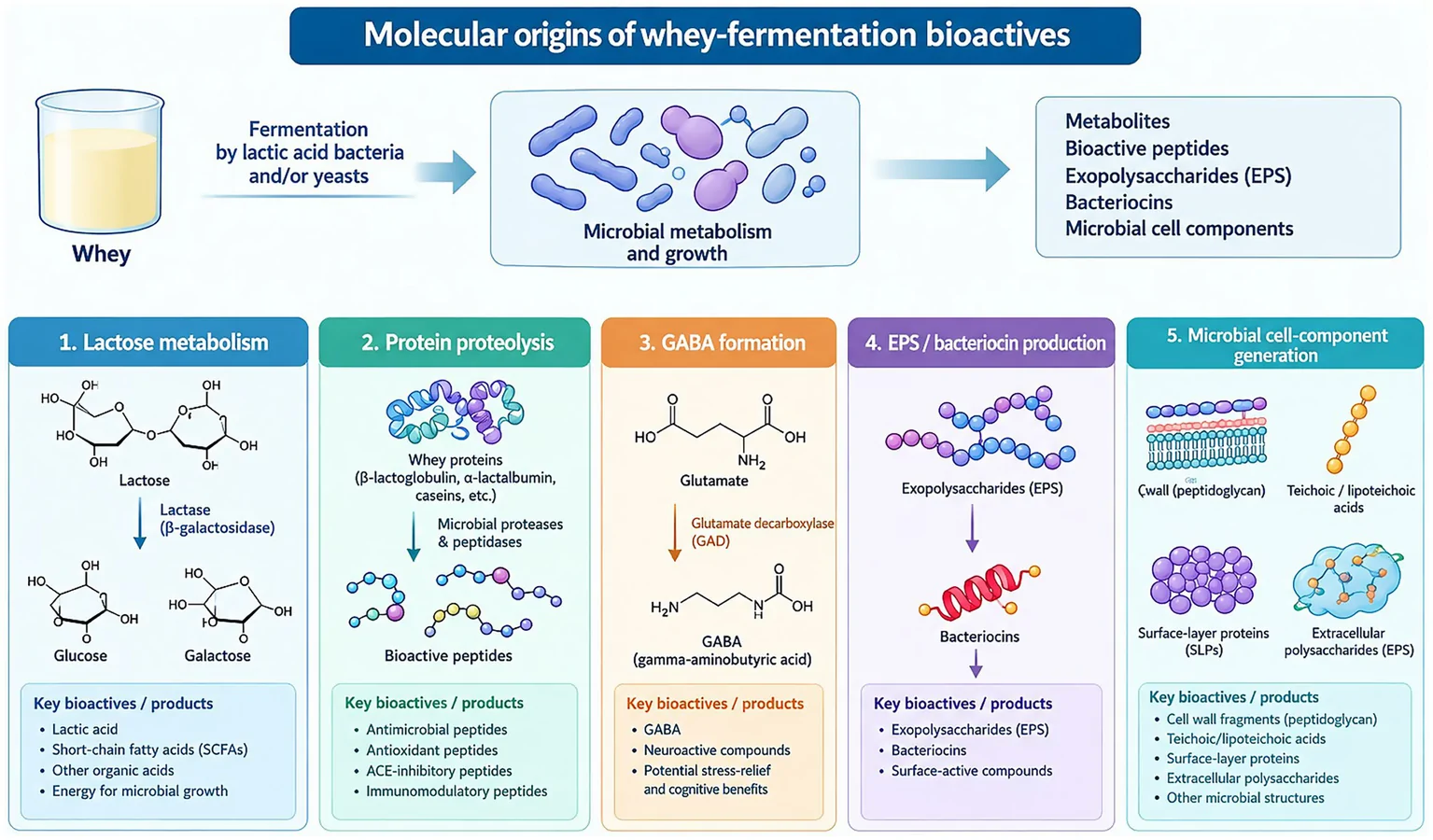
Molecular origins of whey-fermentation bioactives [Created with ChatGPT (OpenAI, GPT-5.5) and edited by the authors]. The schematic figure does not reproduce copyrighted graphical elements. Lactose utilization involves strain-specific transport systems followed by β-galactosidase-mediated hydrolysis and subsequent metabolism of glucose and galactose, primarily toward formation of lactic acid (16, 30, 42, 72, 93, 96), SCFAs (3, 22, 78, 94, 95, 101), and additional organic acids (1, 30, 42, 93, 97). Protein conversion is mediated by the LAB proteolytic system, involving cell-envelope proteinases and intracellular peptidases, resulting primarily in peptides with antimicrobial (36, 73, 76), antioxidant (35, 73, 76, 80), ACE inhibitory (73, 147, 235) or immunomodulatory (36, 146, 147) properties. A separate strain-dependent pathway converts glutamate to γ-aminobutyric acid (GABA) through glutamate decarboxylase (GAD) (114–116, 118, 119). LAB may additionally synthesize extracellular polysaccharides through strain-specific carbohydrate-activated precursor and polymerization pathways (32, 48, 89, 90, 92), whereas ribosomally synthesized bacteriocins are produced through distinct genetic and biosynthetic systems (81, 105, 106, 108, 109, 111, 112). Microbial growth also generates and remodels cell-envelope structures, including peptidoglycan (18, 21, 31), wall and lipoteichoic acids (22, 85, 88), surface-layer proteins (42, 86–88), and extracellular polysaccharides (17, 32, 89–92). Following deliberate inactivation and appropriate characterization, microbial cells and/or their components may contribute to a postbiotic preparation if the current ISAPP definition is met.

### 6.1 Bioactive components generated during whey fermentation

#### 6.1.1 Microbial cellular components contributing to postbiotic preparations

Lipoteichoic acid (LTA), wall teichoic acid (WTA), peptidoglycan, and surface-layer proteins (SLPs) are fundamental structural elements of microbial cell envelopes and key constituents of postbiotic preparations (22). Beyond their structural roles, these molecules exhibit diverse biological activities that underpin the functional and health-promoting effects attributed to postbiotics derived from LAB and related microorganisms (22, 85). The structural properties and anti-inflammatory effects of LTAs isolated from *Lactiplantibacillus plantarum* A3, *Limosilactobacillus reuteri* DMSZ 8533, and *Lactobacillus acidophilus* CICC 6074 have been investigated (85).

Surface-layer proteins (SLPs) from LABs form two-dimensional, self-assembling crystalline lattices composed of protein or glycoprotein subunits, constituting the semi-porous outermost layer of the cell envelope (86, 87). SLPs non-covalently attach to cell wall components, primarily PGN, teichoic acids, lipoteichoic acids, and other acidic or neutral polysaccharides, collectively known as secondary cell wall polymers. Continued structural investigations of these molecules can further support their application in therapeutic and functional food development (85, 88). Translating the knowledge of SLPs gained through laboratory experiments into industrial applications faces hurdles in scalability, mechanistic understanding, and postbiotic compound content standardization (42).

#### 6.1.2 Exopolysaccharides associated with microbial biomass/postbiotic preparations

Microbial exopolysaccharides (EPSs) are high-molecular-weight polymers predominantly composed of monosaccharide residues that are produced and secreted by microorganisms and may remain associated with the cell surface or be released into the extracellular environment (89–91). EPSs can be produced by members of several lactic acid bacterial genera, including *Lactiplantibacillus*, *Lacticaseibacillus*, *Lactococcus*, *Pediococcus*, and *Streptococcus* (90, 92). These bioactive compounds are synthesized by microorganisms under stressful environmental conditions, such as variations in pH, temperature, light intensity, and salinity (32, 90–92). EPS synthesis in LABs is a complex process involving several gene clusters and their corresponding products (90). Glycosyl- and acetyltransferase enzymes are crucial for enhancing EPSs production, thereby representing a potential target for the genetic modification of LAB (17, 90).

The chemical structures of EPSs exhibit significant versatility, ranging from simple linear homopolysaccharides consisting of a single type of monosaccharide to complex branched heteropolysaccharides often substituted with non-sugar components containing acetate, pyruvate, succinate, and phosphate moieties (90). The intricate chemical composition and branching patterns of EPSs, particularly the presence of monosaccharide residues, proteins, molecular branching, and weight, critically influence their biological functions, highlighting the importance of detailed structural characterization (48, 89). Most of the synthesized EPSs are heteropolysaccharides, such as polyhydroxyalkanoate, kefiran, gellan, and xanthan (32, 90, 91), which are typically formed intracellularly (90). The identified gaps in EPS knowledge are related to low yields, complex micro-scale EPS-protein interactions, downstream purification challenges, and the need for standardized genetic engineering and techno-economic scalability (32).

#### 6.1.3 Fermentation-derived microbial metabolites

##### 6.1.3.1 Organic acids

Among the most promising whey valorization methods using LAB fermentation is the production of organic acids (1, 30, 42, 93). Diverse microbial fermentations yield a variety of organic acids, such as lactic, acetic, propionic, butyric, and succinic acids (30, 94). Lactic and succinic acids are categorized under the general group of organic acid compounds, whereas acetic, propionic, and butyric acids are more conveniently recognized as SCFAs (3, 94, 95).

Organic acids derived from whey fermentation primarily possess carboxyl functional groups, conferring acidic properties and water solubility (72, 94). These properties affect microbial inhibition, product separation, and purification (94). The presence of organic acids also contributes to flavor development and preservation in fermented whey products by inhibiting the growth of pathogenic microbes (16, 22, 60, 95).

Lactic acid is the principal organic acid produced during whey fermentation. Its production induces acidification of the fermentation medium, thereby influencing microbial growth dynamics and product stability (42, 93, 96). Homofermentative LAB, including *Lactobacillus delbrueckii* subsp. *bulgaricus*, *Lactobacillus acidophilus*, *Lactobacillus helveticus*, *Lactococcus lactis*, *Lacticaseibacillus casei*, *Lacticaseibacillus paracasei*, and *Streptococcus thermophilus*, predominantly convert sugars into lactic acid with high yields (16, 30, 42, 72, 96). Conversely, heterofermentative LAB, such as *Leuconostoc* and members of the *Lactobacillaceae* family, produce lactic acid alongside acetic acid, ethanol, and carbon dioxide, contributing to flavor complexity (42, 96). Mixed fermentations involving *Lactiplantibacillus plantarum* and *Propionibacterium freudenreichii* (42) or *Lactobacillus delbrueckii* subsp. *bulgaricus* combined with *Streptococcus thermophilus* (72) improve substrate utilization efficiency and broaden the organic acid profile of fermented whey. In addition, mixed cultures combining LAB and yeasts, particularly *Kluyveromyces marxianus*, enhance organic acid profiles and fermentation efficiencies, leveraging yeast thermotolerance and lactose assimilation capability (42, 93).

Succinic acid, a dicarboxylic acid, is generated by strains such as *Actinobacillus succinogenes* (42, 97). This LAB strain efficiently produces succinic acid directly from whey and lactose without enzymatic pretreatment (97). Its production from whey has also gained interest because succinate is a precursor of biodegradable polymers and pharmaceutical compounds (42, 97).

The accumulation of organic acids, particularly lactic acid, during whey fermentation with LAB can inhibit microbial activity and reduce hydrogen production during dark fermentation (94). Strategies to manage acidification, such as pH control without chemical addition, microbial adaptation, and co-digestion, remain areas for development to enhance process stability and economic viability (94). Furthermore, industrial-scale production faces contamination risks, nutrient supplementation costs, and downstream purification complexities, necessitating cost-effective bioprocess designs (42, 97).

##### 6.1.3.2 Short-chain fatty acids (SCFAs)

SCFAs are key bioactive metabolites with diverse biological activities (98–100). They may originate from different biological processes, including microbial fermentation of food substrates and production by the gut microbiota following consumption. SCFAs are characterized by a carbon chain length of fewer than six atoms, with acetate (C2), propionate (C3), and butyrate (C4) as the predominant representatives with biological relevance (3, 95). They are characterized by their volatility, water solubility, and existence in dissociated and undissociated forms (78). According to previous studies (22, 101) LAB, including members of the *Lactobacillaceae* family and *Bifidobacterium* genera, metabolize dietary fiber and other substrates to produce SCFAs. Specific LAB strains that increase SCFAs production include *Lactiplantibacillus plantarum*, *Lacticaseibacillus rhamnosus*, *Lactobacillus acidophilus*, and *Bifidobacterium animalis* (7, 22). Whey is predominantly a lactose- and protein-containing substrate, and LAB fermentation of lactose generally results primarily in lactic acid formation. Acetic acid may also be produced, depending on the microorganism and metabolic pathway involved (16, 42, 94, 96).

In the gastrointestinal tract, SCFAs are primarily produced by the intestinal microbiota through the fermentation of dietary fibers, prebiotics, and other fermentable substrates reaching the colon (102, 103). SCFAs can also be generated during controlled food fermentation processes, including whey fermentation; however, their production and relative abundance depend strongly on the microorganisms, available substrates, and fermentation conditions (3, 95).

A different situation occurs in whey systems supplemented with soluble dietary fibers or prebiotic substrates, such as inulin. The additional fermentable carbohydrate can modify microbial metabolism and enhance the formation of SCFAs, resulting in a profile that differs from that of conventional whey fermentation (3). Accordingly, the SCFA production observed in fiber-supplemented whey systems should be interpreted in relation to the added substrate and microorganisms employed rather than generalized to whey fermentation as a whole. Certain microorganisms, including *Bifidobacterium* and propionibacteria, may contribute to the formation of acetate or propionate, whereas substantial butyrate production is generally associated with specific anaerobic microbial populations and suitable carbon substrates (42, 96). Reports of butyrate formation during cheese-whey dark fermentation should therefore be considered as examples of substrate- and process-specific fermentation rather than evidence that conventional LAB whey fermentation routinely produces butyrate (94).

While acetic acid is often produced concurrently with lactic acid (16, 42, 94, 96) whey supplemented with soluble fibers, such as inulin, significantly enhances SCFA production (3). It has been suggested that *Bifidobacterium animalis* subsp. *lactis* (BPL1) (3, 42) and *Propionibacterium* species (42, 96), produce higher propionic and butyric acid levels than the members of the *Lactobacillaceae* family.

Following consumption, additional SCFAs may be generated in the colon through the fermentation of nondigested or otherwise available substrates by the resident gut microbiota (102, 103). These gut microbiota-derived SCFAs represent a downstream metabolic process and should be distinguished from the SCFAs already present in the fermented whey product. Consequently, the detection of an SCFA in fermented whey does not by itself demonstrate an increase in the corresponding SCFA concentration in the colon or establish a direct gut-health effect (103). The physiological relevance of SCFAs should therefore be discussed separately and in relation to their intestinal availability. Acetate, propionate, and butyrate have been associated with multiple host–microbiota effects, including modulation of intestinal epithelial function, immune responses, and energy metabolism (101); however, such effects should not be attributed directly to SCFAs generated during whey fermentation unless supported by appropriate in vivo or clinical evidence.

Research gaps include the need for strain- and substrate-specific optimization of SCFA production, improved understanding of the metabolic pathways governing SCFA profiles during whey fermentation, and clarification of how fermentation-derived metabolites interact with subsequent gastrointestinal and microbial processes (3, 104). However, scalingup remains challenging because microbial activity, substrate conversion, product recovery, and the stability of individual SCFAs must be controlled under industrial processing conditions (3).

##### 6.1.3.3 Bacteriocins

Bacteriocins are ribosomally synthetized low-molecular-weight peptides or proteins produced by numerous LAB, and are particularly well documented in genera including members of the *Lactobacillaceae* family, and *Lactococcus*, and *Pediococcus* species (105, 106). Recent studies have reported the production of bacteriocins by fermentation using LAB, such as *Lactiplantibacillus plantarum* (106), *Lactococcu lactis* (106), *Pediococcus pentosaceus* (105), *Latilactobacillus sakei* (105)*, Lacticaseibacillus casei* (81), *Pediococcus acidilactici* (81), *Enterococcus faecalis* (105) and *Lacticaseibacillus rhamnosus* ZFM216 (107).

Bacteriocins display considerable structural and molecular diversity, ranging from small peptides less than 5 kDa to larger proteins, and may exhibit linear, cyclic, or post-translationally modified structures (108, 109). Although classification schemes vary slightly among authors, the most widely accepted categorization is into three or four main classes based on molecular structure, molecular weight, mode of action, and genetic characteristics (105, 106, 108, 109). Class I bacteriocins, or lantibiotics, are small, enzymatically modified peptides, generally below 5 kDa, containing characteristic residues such as lanthionine and/or methyllanthionine. Nisin is a well-known example of a Class I bacteriocin. Class II bacteriocins are small, generally heat-stable peptides that undergo no or limited enzymatic modification and are commonly subdivided into pediocin-like bacteriocins, two-peptide bacteriocins, and other linear non-pediocin-like bacteriocins. Class III bacteriocins comprise large, generally heat-labile proteins, often exceeding 10 kDa, and may exhibit bacteriolytic or non-lytic antimicrobial activity. Class IV has traditionally been used for complex bacteriocins containing additional non-proteinaceous components, such as lipid or carbohydrate moieties; however, the validity and boundaries of this class have been debated, as these bacteriocins exhibit activity similar to that of class III.

The composition of the fermentation medium strongly influences bacteriocin production and should provide an adequate supply of carbon and nitrogen sources, vitamins, minerals, and other growth-promoting factors (106, 110). Organic nitrogen compounds, including peptones, yeast extracts, and beef extracts, are commonly employed to stimulate bacterial growth and bacteriocin production (108, 110, 111). The enhancing effect of surfactants is strain-specific and has been reported for strains such as *Lactiplantibacillus plantarum*, *Lactiplantibacillus pentosus*, and *Latilactobacillus sakei* (110–112). Carbon sources such as glucose, sucrose, and lactose are commonly preferred because of their rapid uptake and efficient metabolism, which directly supports bacterial growth and metabolite production (108, 110). In contrast, complex carbohydrates may result in lower bacteriocin production than readily metabolizable sugars under certain fermentation conditions (110). Optimal production temperatures are generally strain-dependent and frequently fall within the range of approximately 30–37 °C, although maximum bacteriocin production may occur at lower or suboptimal growth temperatures in some strains (110). In addition, bacteriocin production is highly pH-dependent and often peaks under mildly acidic conditions (pH 5.5–6.0). Although LAB are facultative anaerobes and generally aerotolerant, oxygen availability may further influence their growth, metabolism, and bacteriocin expression, reflecting the strain-dependent oxygen tolerance and regulatory responses of LAB (108, 110).

Despite their considerable potential as natural antimicrobial agents, the practical application of bacteriocins, particularly for therapeutic use, is influenced by several factors, including their spectrum of activity, stability, susceptibility to proteolysis, bioavailability, safety, pharmacokinetic properties, and regulatory status (105, 109, 113). Consequently, although nisin has an established history of use as a food preservative, the broader therapeutic application and oral administration of bacteriocins require further characterization and validation, including appropriate safety and clinical studies (109).

##### 6.1.3.4 γ -Aminobutyric acid (GABA)

GABA is a non-proteinogenic four-carbon amino acid that contains an amino group on the gamma carbon, distinguishing it from standard alpha amino acids (114). It is highly soluble in water because of its polar groups and is stable under acidic conditions. GABA is a zwitterionic molecule at physiological pH, which aligns with the optimal pH range (4.5–6.0) for glutamate decarboxylase (GAD) activity during fermentation (114–116). The production of GABA by LAB involves the enzymatic decarboxylation of glutamate catalyzed by GAD, which requires pyridoxal-5′-phosphate (PLP) as a cofactor (114, 116, 117). Whey itself contains limited free glutamate; thus, supplementation with glutamate (114, 115) and yeast extracts (116, 118) enhances the GABA yield.

LAB form genera, such as *Lactiplantibacillus*, *Lactococcus*, *Leuconostoc*, *Pediococcus*, and *Enterococcus*, have been reported as primary microbial producers of GABA during whey fermentation (114, 116, 119). Among LAB, *Lactiplantibacillus plantarum* and *Levilactobacillus brevis* are frequently reported as high GABA producers (67, 114, 115), while *Enterococcus malodoratus* has been identified as a promising GABA producer in whey-based beverage fermentation (119). The production of GABA by LAB involves the enzymatic decarboxylation of glutamate catalyzed by glutamate decarboxylase (GAD), which requires pyridoxal-5′-phosphate (PLP) as a cofactor (116, 117). Whey itself contains limited free glutamate; thus, supplementation with glutamate (114, 115) and yeast extracts (117, 118) enhances GABA yield. Mixed cultures, including yeasts such as *Kluyveromyces lactis* with LAB, have been shown to improve GABA production during whey fermentation by modulating environmental factors and metabolic interactions (5). Specifically, fermented whey beverages produced by LAB, such as *Pediococcus pentosaceus* ENM104 and *Lactiplantibacillus plantarum* SPS109, exhibited increased GABA content (118). Challenges in scaling up fermentation processes using whey, including controlling contamination, maintaining culture viability, and ensuring consistent GABA production, remain to be addressed for commercial feasibility (30, 114).

##### 6.1.3.5 Phenolic-associated compounds

Phenolic-associated compounds (PAC) are not considered major intrinsic constituents of pure whey (120). Their presence in whey-based fermented products is associated with minor phenolic constituents naturally transferred from milk and dietary-derived compounds (121), or, particularly, the incorporation of plant-derived ingredients, such as fruits, fruit extracts, herbs, or other plant materials (121, 122). The incorporation of fruits and other plant-derived materials introduces phenolic compounds into whey-based formulations, while subsequent LAB fermentation may modify their concentration, chemical form, extractability, and bioaccessibility through matrix disruption and microbial biotransformation (123). PAC in whey fermented contribute significantly to the bioactive profile, functional properties, and sensory attributes of fermented whey products (3, 121, 122).

Phenolic-associated compounds in fermented whey products can arise through three interconnected mechanisms: plant-derived contribution, whereby fruits or other plant materials introduce phenolic compounds into the whey matrix (120, 121, 123), fermentation-mediated transformation, whereby LAB release, hydrolyze, degrade, or otherwise biotransform available phenolic substrates and may produce related microbial metabolites such as phenyllactic acid (PLA) (124), and matrix-interaction effects, whereby phenolics interact with whey proteins and other components, affecting their stability, release, extractability, and bioaccessibility (120, 122, 125). According to the third mechanism, whereby plant-derived phenolic compounds interact with whey proteins through non-covalent and, under certain conditions, covalent interactions, potentially modifying protein conformation and phenolic profile and properties (126) These interactions modify the secondary and tertiary structures of whey proteins, influencing their allergenicity potential, thermal stability, and surface hydrophobicity (121, 122, 125, 127).

For example, in fermented ewe's milk whey–fruit smoothies, whey primarily serves as the protein-rich dairy matrix, whereas the fruit component is the principal source of phenolic compounds and anthocyanins (120). Consequently, the phenolic profile of a fermented whey–plant formulation reflects the combined contributions of the original plant material, fermentation-mediated transformations, and interactions between phenolics and components of the whey matrix.

Among PACs, phenyllactic acid (PLA) has frequently been detected at appreciable concentrations in LAB-fermented food systems and has been associated with antimicrobial activity (124, 128). It is a phenyl-derived organic acid and a microbial metabolite (124), and its formation during LAB fermentation is linked to the microbial metabolism of aromatic amino acids, particularly phenylalanine (124). Plant-derived phenolic compounds encompass structurally diverse molecules ranging from low-molecular-weight phenolic acids to flavonoids and more complex polyphenols (124, 129), including phenolic acids, such as gallic, vanillic, caffeic, syringic, p-coumaric, ferulic, chlorogenic, and salicylic acids, which also contribute to the antimicrobial and antioxidant properties of fermented whey (129). During LAB fermentation, these compounds may undergo release, degradation, or biotransformation rather than simple de novo synthesis. LAB can metabolize phenolic substrates through several enzymatic pathways, including glycosidase-, esterase-, decarboxylase-, and reductase-mediated reactions (123). Such transformations may convert glycosylated or esterified phenolics into more readily extractable forms or alternatively lead to the formation of structurally different microbial metabolites.

PLA and other low-molecular-weight phenolic acids (<200 g mol^-1^) are highly effective antimicrobial agents, as supported by molecular docking and quantitative structure-activity relationship (QSAR) analyses (130). Their antioxidant capacity, largely attributed to their ability to donate hydrogen atoms and scavenge free radicals (3), is due to the presence of conjugated double bonds (130). PACs also contribute to sensory attributes and shelf-life extension when incorporated into fermented whey products (124, 130).

Among LAB species, *Lactiplantibacillus plantarum* has been reported to be the most prominent PLA producer among the tested strains (124), whereas *Pediococcus acidilactici* and a mixture of *Latilactobacillus sakei* and *Staphylococcus xylosus* yielded diverse phenolic compound (PC) profiles with significant antioxidant activities (131). In addition to these, other bacteria, including *Enterococcus faecium*, *Pediococcus pentosaceus*, and *Pediococcus acidilactici*, have been identified as potent producers of PCs and other postbiotic metabolites with antimicrobial and antioxidant properties (132).

##### 6.1.3.6 Biosurfactants

Biosurfactants derived from whey fermentation represent a promising class of bioactive compounds with versatile applications in food technology owing to their biodegradability, low toxicity, and multifunctional applications (133). Utilizing whey not only valorizes it but also supports sustainable and cost-effective biosurfactant production processes that exhibit desirable physicochemical and bioactive characteristics (84, 134–136). Biosurfactants belong to several chemical classes, including glycolipids, lipoproteins, glycolipoproteins, and glycoproteins (137, 138). They contribute to the emulsification, foaming, texture enhancement, and shelf-life extension of food products (138). The emulsifying and antimicrobial properties of biosurfactants are attributed to their amphipathic molecular structure, which enables them to reduce surface and interfacial tension, disrupt microbial membranes, and inhibit biofilm formation (133). Whey-derived biosurfactants, particularly lipopeptides, exhibit functional resilience and remain active at extreme temperatures and across a wide pH range, making them suitable for incorporation into food-packaging materials and pharmaceutical formulations (84, 136). Emerging non-thermal inactivation technologies preserve biosurfactant bioactivity during downstream processing more effectively than conventional thermal methods, improving their stability and shelf life (84).

During whey fermentation, selected LAB can produce cell-bound or excreted biosurfactant compounds with heterogeneous chemical compositions, including glycolipids, glycoproteins, and glycolipoprotein biosurfactants (137). However, the production of these metabolites depends strongly on the producer microorganism and fermentation conditions, while bacteria utilize lactose, proteins, and minerals in whey to synthesize biosurfactants (134, 138). The addition of sugars, proteins, and minerals can further enhance biosurfactant-related biomass production (133). For example, *Lactiplantibacillus plantarum* ATCC 8014 was used for ultrasound-assisted production of biosurfactants with glycolipoprotein structures using whey permeate combined with sugarcane molasses and yeast, with enhanced yield and antiviral activity (135). Additionally, *Lacticaseibacillus paracasei* strain was reported as a lactic microbial producer of glycolipoprotein biosurfactants assisted by supplementation with molasses cane sugar (137). Glycoprotein biosurfactants can be produced during whey fermentation using bacterial strains such as *Lacticaseibacillus casei* 8/4 and *Ligilactobacillus agilis* CCUG31450 (137). Glycolipid biosurgactants were produced by whey fermentation with *Lactobacillus helveticus* and additional yeast supplementation (139). A recent study on the fractionation and chemical characterization of cell-bound biosurfactants produced by whey fermentation with *Limosilactobacillus fermentum* strain suggested the formation of both glycoprotein and glycolipid biosurfactants (140).

The surface-active properties of biosurfactants in SFSs are typically assessed by measuring the critical micelle concentration and emulsification index, which indicate the functional efficacy of the extracts (133–135). Further development of multi-omics analyses coupled with artificial intelligence (AI) and machine learning (ML) can facilitate the discovery of novel biosurfactants, strain improvement, predictive modeling of tailored biosurfactant production, and scale-up that meet specific food industry requirements and functionality (138, 141).

##### 6.1.3.7 Vitamins

Vitamins are considered important microbially synthesized metabolites because they remain in the fermented matrix or CFS after the removal of bacterial cells and may contribute to the improved nutritional value and biological activity of fermented products (6, 142). Whey contains lactose, peptides, minerals, and residual proteins that support LAB growth and metabolic activity, enabling the selected strains to synthesize B-group vitamins, especially folate (vitamin B9), thiamine (vitamin B1), riboflavin (vitamin B2), cobalamin (B12), pyridoxine (B6), and other B vitamins, as well as vitamin K (6, 42, 79, 142, 143). Vitamins play crucial roles in human health by acting as cofactors in enzymatic processes and contributing to energy metabolism, gene regulation, and immune modulation (79, 142). While vitamin K and most B-group vitamins can be synthesized by intestinal bacteria and probiotic microorganisms, folate is mainly formed during the fermentation process by LAB and other bacterial strains (79, 95). The reported strains capable of folate production include those from the *Lactobacillaceae* family, and genera, such as *Lactococcus*, *Streptococcus*, and *Bifidobacterium* (79, 100, 142). Riboflavin (vitamin B2) is a precursor of flavin mononucleotide and flavin adenine dinucleotide, which are essential cofactors in redox reactions, energy metabolism, and cellular antioxidant defense systems (79, 142). Thiamine serves as a coenzyme in crucial metabolic processes that aid the development of functional microorganisms grown on whey and supports microbial viability by facilitating essential cellular activities for growth and adaptation (42). Although LAB are not pronounced cobalamin producers, certain strains, particularly *Limosilactobacillus reuteri* (42), *Lactococcus lactis* E32, *Levilactobacillus brevis* G31, and *Pediococcus pentosaceus* L51 (144), have demonstrated the capacity to synthesize pseudo cobalamin under specific growth conditions.

#### 6.1.4 Bioactive peptides

Bioactive peptides (BAPs) derived from fermented whey constitute an important group of bioactive postbiotic compounds generated during the microbial proteolysis of whey proteins, which are important precursors for BAP formation (36, 38, 54, 145). Alternatively, they can be obtained by chemical hydrolysis using commercial enzymes, such as proteases, exopeptidases, and endopeptidases (36, 38, 54, 146). This technique offers certain benefits for producing BAPs compared to other methods, including a significant increase in the reaction rate (38, 54).

BAPs are short molecule sequences, usually containing 2-50 amino acid residues, which are chemically classified by chain length (short chain, medium chain, and long chain peptides with respective length of 2-10, 10-20, and over 20 amino acids), molecular weight (dipeptides and tripeptides, oligopeptides, and polypeptides with respective mass ranges of <0.5 kDa, 0.5-3.0 kDa, and >3.0 kDa), charge (anionic and cationic), hydrophobicity, and secondary structure. These chemical characteristics directly dictate the interactions between peptides and biological targets (38, 54).

Microorganisms such as *Lactobacillus helveticus*, *Lacticaseibacillus casei*, *Lactiplantibacillus plantarum*, and *Lactococcus lactis* are highly valued for their ability to break down whey proteins and produce bioactive peptides (19, 36, 145). Specifically, the utilization of *Pediococcu acidilactici* SDL1414, *Lactobacillus helveticus*, *Lactiplantibacillus plantarum* QS670, and *Lactiplantibacillus rhamnosus* JDFM6 (36, 145) for whey fermentation was associated with the production of peptides with pronounced ACE inhibitory activity. In addition, fermentation processes conducted with *Streptococcus thermophilus* SY-102 and *Lacticaseibacillus rhamnosus* GG, both separately and together, demonstrated the possibility of producing ACE inhibitory peptides (147). Whey fermentation with *Lactiplantibacillus plantarum*, *Streptococcus thermophilus*, and *Lactobacillus acidophilus* LA-5 yields peptides with fractions under 1kDa, that exhibit enhanced antioxidative and antimicrobial activities compared to larger peptides (31, 36, 38, 145). The main constraints observed during peptide production by whey fermentation were the optimization of the production processes to obtain higher yield and specificity while ensuring enhanced peptide stability and bioavailability.

Table 4 presents a summary of various studies investigating the generation of bioactive peptides derived from whey and whey protein substrates through microbial and enzymatic fermentation processes. It highlights the different microorganisms used, fermentation conditions, and the resulting bioactive properties, such as angiotensin-converting enzyme (ACE) inhibition, antioxidant activity, and antimicrobial effects. The peptide fractions accumulated after fermentation varied in molecular weight, with notable bioactivity often associated with low molecular weight peptides.

**Table 4:** Example studies on the generation of whey-derived bioactive peptides during microbial and enzymatic fermentation.

Whey/protein substrate	Microorganism(s)	Fermentation conditions	Main findings	Peptide fractions*	References
Whey	Enzymatic fermentation with bromelain; no additional microorganisms were added	Bromelain hydrolysis at 55 °C, pH = 7.0, 1 h with HIUS	Enhanced enzymatic hydrolysis modified the peptide profile, resulting in increased production of ACE-inhibitory peptides	>10 kDa, 5–10 kDa, 3–5 kDa, 1–3 kDa, and <1 kDa. The 1–5 kDa fractions showed strong ACE inhibition	(235)
Dairy sludge (waste whey)	Co-culture: *Limosilactobacillus fermentum* 4–17 + *Saccharomyces cerevisiae* PTCC 5052	34.86 °C, 43.1 h, simultaneous inoculation	High wet biomass production; ACE inhibitory activity; antioxidant activity DPPH antimicrobial activity against *S. aureus*	Not specified	(73)
Whey protein concentrate	*Streptococcus thermophilus* SY-102	42 °C, 36 h, pH=7.5, after enzymatic hydrolysis with Proteinase K	High ACE inhibition due to robust proteolytic system and release of potent tripeptide	Peptides <1.4 kDa accumulated after fermentation	(147)
Whey protein concentrate	*Lacticaseibacillus rhamnosus GG*	42 °C, 6.75 h, pH=7.5, after enzymatic hydrolysis with Proteinase K	Moderate ACE inhibition; higher concentration of free amino groups	Peptides <1.4 kDa accumulated after fermentation	(147)
Whey protein concentrate	Mixed culture: *Streptococcus thermophilus SY-102 + Lacticaseibacillus rhamnosus GG*	42 °C, 36 h, pH=7.5, after enzymatic hydrolysis with Proteinase K	Low ACE inhibition due to over- hydrolysis and antagonistic interactions	Peptides <1.4 kDa accumulated after fermentation	(147)
Whey protein concentrate	*L. delbrueckii* subsp. *Delbrueckii LbL2*	37 °C, pH=6.5, 72 h	Moderate protein hydrolysis, high DPPH radical scavenging and low FRAP	< 3kDa (biomass content 1.05 mg/mL)	(35)
Whey protein concentrate	*L. delbrueckii* subsp. *Bulgaricus LbL9*	72 h, 37 °C, pH=6.5	Moderate protein hydrolysis, high DPPH radical scavenging and low FRAP	< 3kDa (biomass content 0.58 mg/mL)	(35)
Whey protein concentrate	*Lacticaseibacillus paracasei* AF43	72 h, 30 °C, pH=6.5	Low protein hydrolysis, high DPPH radical scavenging and moderate FRAP	< 3kDa (biomass content 0.39 mg/mL)	(35)
Whey protein concentrate	*Lactiplantibacillus plantarum* 297	72 h, 30 °C, pH=6.5	Low protein hydrolysis, high DPPH radical scavenging and moderate FRAP	< 3kDa (biomass content 0.47 mg/mL)	(35)
Whey protein concentrate	*Streptococcus macedonicus LbL43*	72 h, 30 °C, pH=6.5	Low protein hydrolysis, high DPPH radical scavenging and high FRAP	< 3kDa (biomass content 0.49 mg/mL)	(35)
Whey protein concentrate	*L. delbrueckii* subsp. *lactis* LbL33	72 h, 30 °C, pH=6.5	Moderate protein hydrolysis, high DPPH radical scavenging and moderate FRAP	< 3kDa (biomass content 0.62 mg/mL)	(35)

*Peptide fractions, accumulated peptide fractions after fermentation; HIUS, high intensity ultrasound; DPPH *-* 2,2-diphenyl-1-picrylhydrazyl; FRAP *-* ferric reducing antioxidant power; ACE, angiotensin-converting enzyme.

Despite its advantages, microbial fermentation has limitations, including relatively low peptide yields and diminished specificity in peptide generation compared to enzymatic hydrolysis. Batch-to-batch variability and potential consumption of peptides by microbes during fermentation also pose challenges for industrial scalability (36). Recent advances include solid-state fermentation (SSF) using selected microorganisms, which can simultaneously produce proteases and bioactive peptides from food by-products, such as whey (36, 80).

### 6.2 Characterization and determination of bioactive compounds in postbiotic preparations from fermented whey

The complex composition of postbiotic preparations formed during whey fermentation requires the application of multiple complementary analytical techniques capable of identifying and quantifying the bioactive molecules. Because postbiotics-related compounds represent a heterogeneous mixture of low- and high-molecular-weight compounds, a single analytical approach is insufficient for their complete characterization. Consequently, modern postbiotic preparations research increasingly relies on integrated analytical characterization that mainly combine chromatographic, spectroscopic, electrophoretic, microbiological, and omics-based approaches to achieve comprehensive qualitative and quantitative analyses (148)

Colorimetric methods for postbiotics derived from fermented whey are rapid, cost-effective, and high-throughput analytical techniques used to quantify bioactive metabolites, such as peptides, organic acids, and phenolic compounds, at a screening level (3, 70). For example, enzymatic ultraviolet (UV) spectroscopy has been used as a rapid qualitative screening tool for the presence of GABA in fermented dairy products (5, 117). In addition, UV spectroscopy is the method of choice for quantifying the total phenolic content in whey fermentation supernatants and protein-polyphenol conjugates using the Folin–Ciocalteu method (3, 68, 127, 131, 132).

Chromatographic techniques are the cornerstone for profiling and quantifying volatile and nonvolatile postbiotic-related compounds. EPSs are quantified using gas chromatography (GC) or high-performance liquid chromatography (HPLC) with a refractive index detector, by analyzing the sugar monomers after EPSs hydrolysis (88, 149). HPLC is also used to quantify organic acids in whey fermentation products and remains the gold standard for their analysis (3, 70). GC with flame ionization detection (FID) or mass spectrometry (MS) is particularly used for SCFAs analysis after derivatization (150). Additionally, GC and HPLC coupled with mass spectrometry are used for the separation, identification, and quantification of biosurfactant components, including lipopeptides and glycolipids (136). In addition to HPLC and GC, thin-layer chromatography is used as a rapid, low-cost qualitative screening tool for the presence of GABA (115, 117) and other postbiotic compounds (15, 18) before employing quantitative methods. However, HPLC with UV or fluorescence detection is the most commonly used technique for GABA quantification (5, 115).

Peptides and bacteriocins are typically separated and purified using size-exclusion chromatography, which allows the fractionation of peptide and bacteriocin mixtures with varying activities based on hydrophobicity and molecule size, followed by quantification using analytical HPLC (36, 151). Furthermore, peptide and bacteriocin profiling can be conducted using electrophoretic methods, such as sodium dodecyl sulfate–polyacrylamide gel electrophoresis (SDS–PAGE) or tricine-SDS-PAGE, which are particularly employed for molecular weight estimation and purity assessment, especially for small-molecule peptides (3.5–5 kDa) (64, 151).

Standalone spectroscopic techniques are often used to provide in-depth structural insights into postbiotic-related compounds (7). For example, Fourier-transform infrared spectroscopy (FTIR) and nuclear magnetic resonance (NMR) analyses have revealed structural heterogeneity among LTAs, reflecting the distinct adhesion properties of each strain (85). Similarly, the structural characterization of WTAs from *Lactiplantibacillus plantarum* was performed using ^1^H- and ^13^C-NMR spectroscopy (88). Methods such as scanning electron microscopy (SEM) combined with atomic force microscopy have been employed to visualize SLPs on whole *Lactobacillaceae* cells, whereas transmission electron microscopy has been used to analyze isolated cell wall fragments (87). The above-mentioned spectroscopic techniques detect the presence of characteristic functional groups that are fingerprints for postbiotic-related compounds identification (121, 127, 132, 149). Specifically, these techniques have been used to characterize structural changes and functional groups, such as in protein-polyphenol complexes, and to identify morphological changes in whey protein structures after conjugation with PACs (125, 126).

The structural identification of postbiotic-related compounds is currently dominated by LC coupled with tandem mass spectrometry (LC–MS/MS), particularly high-resolution (HR)MS, which is a valuable tool for targeted and untargeted postbiotic-related compounds analysis. Specifically, the application of these techniques expands the coverage of low-abundance peptides, bacteriocin fractions, and EPSs monomers after hydrolysis (39, 54, 83). Tandem MS and HRMS enable accurate mass determination, molecule fragmentation, and identification of biomarker moieties associated with the health-promoting properties of bioactive molecules (9, 83). HRMS is a key technology used to profile PACs in fermented whey, enabling the precise fragmentation pattern analysis of a broad range of molecules (128). Vitamin characterization by combining the separation power of LC with the detection specificity of MS enables the precise identification and quantification of vitamins, including their isomers and metabolites, in complex whey fermentation products (79).

Owing to the powerful properties of MS and HRMS, omics-based analysis has become one of the most advanced analytical approaches for the comprehensive characterization of postbiotic preparations generated during whey fermentation by LAB (104, 152). Unlike targeted analyses that quantify predefined metabolites, metabolomics aims to identify and quantify the complete set of low-molecular-weight compounds (<1,500 Da) in biological matrices (78). This technique provides an effective strategy for obtaining an integrated chemical fingerprint of postbiotic preparations in fermented whey (127).

The combination of metabolomics, proteomics, glycomics, lipidomics, and bioinformatics enables a systematic understanding of whey fermentation and the identification of novel bioactive compounds (84, 138, 153). Proteomic and peptidomic profiling using MS combined with bioinformatics and *in silico* prediction tools can support the identification and functional annotation of bioactive peptides, helping elucidate their structure–activity relationships (36, 38, 151). Furthermore, HRMS supported by artificial intelligence, machine learning, and chemometric modeling is expected to facilitate the discovery of robust postbiotic-related biomarkers, enhance strain selection, improve process monitoring, and develop tailored postbiotic preparations to support industrial requirements for standardization (84, 136, 148).

## 7 Bioactivity of postbiotic compounds and health benefits

Recent whey-focused reviews reinforce this framing by showing that whey and whey-protein ingredients used in fermented foods can generate bioactive peptides, bacteriocins, exopolysaccharides, organic acids, phenolic compounds, flavonoids, and enzymes with antioxidant, antibacterial, anti-inflammatory, immunomodulatory, and metabolic effects on the body. This supports the interpretation of fermented whey as both a fermentation substrate and a source of biologically active whey-derived compounds, rather than a passive carrier matrix.

The main bioactive compound groups generated or retained in fermented whey-derived postbiotic-associated preparations, along with their proposed mechanisms and functional relevance, are summarized in Table 5.

**Table 5:** Main bioactive compound groups in fermented whey-derived postbiotic-associated preparations and their potential functional relevance.

Bioactive compound	Main origin in fermented whey	Main bioactivity	Proposed mechanism(s)	References
Whey-derived bioactive peptides	Proteolysis of β-lactoglobulin, α-lactalbumin, lactoferrin, glycomacropeptide and other whey proteins during fermentation/digestion	Antioxidant, antimicrobial, ACE-inhibitory, immunomodulatory	ROS scavenging, metal chelation, membrane disruption, ACE inhibition, cytokine modulation	(7, 35, 118, 155)
Organic acids	LAB and bifidobacterial fermentation of lactose and other substrates	Antimicrobial, gut-relevant, preservation	pH reduction, intracellular acidification, enzyme inhibition, membrane stress	(3, 28, 51)
Short-chain fatty acids	Direct production by selected strains or fibre-supplemented whey fermentation; possible gut microbial cross-feeding	Gut barrier support, anti-inflammatory potential, metabolic signalling	Colonocyte energy supply, GPCR signalling, HDAC inhibition, Treg modulation	(3, 98, 99, 237)
Bacteriocins and bacteriocin-like substances	LAB fermentation, especially selected protective or starter cultures	Antimicrobial, antibiofilm, food safety	Pore formation, membrane disruption, inhibition of sensitive pathogens	(15, 28, 98)
Exopolysaccharides	EPS-producing LAB during fermentation	Antioxidant, anti-inflammatory, gut barrier support, antibiofilm	ROS reduction, adhesion interference, tight-junction support, cytokine modulation	(7, 98, 99, 162)
Phenolic-associated compounds and transformed minor constituents	Release/transformation during fermentation; substrate-dependent	Antioxidant, antimicrobial support	Radical scavenging, redox modulation, possible pathogen inhibition	(68, 70, 132)
Inactivated microbial cells and cell-wall components	Heat treatment, drying, sonication or other post-fermentation processing	Immunomodulatory, anti-inflammatory, gut barrier support	Interaction with PRRs such as TLRs and NOD-like receptors, cytokine modulation	(24, 99, 154, 238)
GABA and other low-molecular-weight metabolites	Strain-dependent LAB metabolism during whey fermentation	Gut–brain-axis relevance, metabolic support	Neuroactive signalling potential, indirect gut-associated effects	(67, 118)
Biosurfactants and volatile antimicrobial metabolites	LAB metabolism and cell-associated compounds	Antimicrobial, antibiofilm	Reduced adhesion, membrane interaction, biofilm disruption	(84, 154, 159)

### 7.1 Antioxidative properties

The antioxidant potential of fermented whey should not be attributed to a single compound class, but rather to the combined contribution of whey-derived peptides, soluble microbial metabolites, exopolysaccharides, organic acids, phenolic-associated compounds, and, in some preparations, inactivated microbial cells or cell wall fragments (98, 99, 154). Available evidence indicates that fermented whey and whey-derived postbiotic-associated preparations can show antioxidant capacity in chemical assays and antioxidant potential in cellular or food-matrix models. However, these effects remain strain-, substrate-, and process-dependent, and future studies should combine antioxidant assays with peptide profiling, metabolomics, food-matrix validation, and appropriate biological or clinical evaluation.

A central mechanism behind the antioxidative properties of fermented whey is the release of bioactive peptides from whey proteins during microbial proteolysis. These peptides contribute to antioxidant activity by scavenging reactive oxygen species, chelating transition metals and supporting endogenous antioxidant pathways. In this context, fermented whey should be viewed not only as a carrier of microbial metabolites, but also as a substrate for the generation of whey-protein-derived antioxidant peptides (28, 155). This peptide-based interpretation is further supported by another study that reviewed whey proteins and their derivatives as sources of functional ingredients with antioxidant, antimicrobial, immunomodulatory, and gastrointestinal-support properties (156).

Experimental evidence from fermented whey studies supports this interpretation. A functional fermented whey beverage produced using *Pediococcus pentosaceus* ENM104 and *Lactiplantibacillus plantarum* SPS109 showed a marked increase in oxygen radical absorbance capacity after fermentation (118). The fermented whey reached an ORAC value of 362.06 ± 8.63 nmol TE/mL after 72 h, suggesting that fermentation improved the antioxidant profile of the whey matrix. The authors attributed this increase partly to the antioxidant peptides and LAB-derived antioxidant molecules generated during fermentation.

Further evidence has emerged from whey-based postbiotic production studies. Whey-based media supplemented with soluble fibres, including inulin and chia mucilage, were evaluated for the production of cell-free postbiotic supernatants by *Lactiplantibacillus plantarum* 299v, *Lacticaseibacillus casei* Shirota, and *Bifidobacterium animalis* subsp. *lactis* BPL1 (3). The study showed that whey media supported probiotic growth and generated postbiotic supernatants with measurable antioxidant activity. In particular, soluble fibre supplementation significantly improved the antioxidant activity of *L. plantarum* 299v postbiotics, as measured by DPPH and KMnO₄ agar assays. These findings indicate that whey-based antioxidant activity can be modulated by strain selection and substrate formulation.

A study reported that a whey model beverage fermented using 12 LAB strains isolated from raw milk dairy products provided additional direct evidence (35). Fermentation resulted in strain-dependent protein hydrolysis, accumulation of low-molecular-weight peptides, and improved antioxidant activity of the fermented product. In particular, *L. delbrueckii* subsp. *lactis* LbL37 increased DPPH radical-scavenging activity by approximately 40% compared to that of the unfermented control, whereas *Streptococcus macedonicus* LbL43 exhibited the highest ferric-reducing power.

The postbiotics from *Lactobacillus acidophilus* LA-5 produced in conventional MRS broth and UF cheese whey were compared for their antioxidant activity (70). Both preparations showed antioxidant potential, although MRS-derived postbiotics had stronger DPPH and ABTS radical-scavenging activity than cheese whey-derived postbiotics. Nevertheless, cheese whey-derived postbiotics also increased the total antioxidant capacity of HCT-116 cell supernatants in a concentration-dependent manner, indicating that whey-based postbiotic preparations can retain biologically relevant antioxidant activity.

Broader LAB postbiotic studies support the involvement of phenolic-associated compounds, low-molecular-weight metabolites, EPS and soluble microbial fractions in antioxidant activity (68, 131, 132, 157). Mechanistically, these effects involve radical scavenging, metal chelation, ROS reduction, inhibition of lipid peroxidation and support of antioxidant enzyme systems, including catalase, superoxide dismutase and glutathione-related pathways (98, 99, 158).

The available evidence indicates that fermented whey and whey-derived postbiotic-associated preparations may possess meaningful antioxidant potential. However, antioxidant effects remain strain-, substrate-, and process-dependent, and future studies should combine antioxidant assays with peptide profiling, metabolomics, and food-matrix or biological validation.

### 7.2 Antimicrobial activity

Unlike conventional antimicrobial agents, which are typically based on single active compounds, fermented whey represents a complex matrix in which several antimicrobial mechanisms can act simultaneously. These include environmental acidification, disruption of microbial membranes, interference with intracellular pH homeostasis and energy metabolism, inhibition of cell wall synthesis, reduction of pathogen adhesion, inhibition of biofilm formation, and modulation of the surrounding microbiota. Therefore, the antimicrobial activity in fermented whey should be interpreted as a multifactorial and strain-dependent property shaped by the starter culture, whey composition, fermentation conditions, and post-fermentation processing (98, 99, 154, 159).

Organic acids are among the most important antimicrobial compounds in whey fermentation. LAB fermentation of whey commonly generates lactic and acetic acids, and in some cases, citric, propionic, or butyric acids also contribute to antimicrobial activity. These acids can lower pH, inhibit non-acid-tolerant microorganisms, penetrate microbial cells in an undissociated form, dissociate intracellularly, and disrupt pH homeostasis, enzyme activity, and energy gradients. Several studies (79, 84, 99) have similarly described organic acids and SCFAs as postbiotic compounds with multitarget antimicrobial mechanisms, including acidification, membrane permeabilisation, intracellular acid stress, and redox imbalance.

The antimicrobial relevance of whey fermentation is strongly supported by whey-focused reviews. Whey and other dairy waste streams have been described as suitable substrates for LAB fermentation and the production of antimicrobial compounds, including bacteriocins, bacteriocin-like inhibitory substances, antimicrobial peptides, organic acids, and enzymes (28). The review also notes that whey-based substrates have been used to produce anti-*Listeria* compounds in previous studies. This is important because many LAB bacteriocins, including nisin- and pediocin-like compounds, are particularly effective against gram-positive foodborne pathogens, such as *Listeria monocytogenes*.

Several Class I and II LAB bacteriocins have been reported to be heat- and pH-stable, indicating that antimicrobial activity may persist after heat-based postbiotic preparation (98).

Direct fermented whey evidence was provided in a study where the authors developed a functional fermented whey beverage using *Pediococcus pentosaceus* ENM104 and *Lactiplantibacillus plantarum* SPS109 (118). The fermented whey inhibited *Bacillus cereus*, *Listeria monocytogenes* and *Streptococcus mutans*, with MIC values ranging from 30–40%. It also showed antibiofilm activity, with biofilm inhibitory concentrations of 35–40%, and the strongest effect was observed against *S. mutans*, where biofilm inhibition reached 98.52 ± 0.99%.

Additional direct evidence from whey fermentation demonstrated that several LAB-fermented whey beverages inhibited pathogenic bacteria, particularly *Listeria monocytogenes* (35). This supports the idea that antimicrobial activity in fermented whey can be linked to selective proteolysis and peptide release, in addition to acidification and other fermentation metabolites.

Similarly, postbiotics from *Lactobacillus acidophilus* LA-5 produced in UF cheese whey showed stronger antimicrobial activity against *L. monocytogenes* and *E. coli* than those produced in conventional MRS broth (70). This effect was associated with the higher organic acid profile of the whey-derived preparation, especially lactic, citric, and butyric acids.

Several dairy and food-matrix studies support the bioprotective potential of LAB-derived postbiotics, including inhibition of foodborne pathogens in pasteurised milk, meat products and poultry matrices (68, 112, 131, 132, 157, 160). Fermented whey has also been investigated as a natural coating for the biopreservation of cheese (161). Although some of these studies are not whey-specific, they reinforce the need to validate postbiotic antimicrobial activity in real food systems rather than relying only on agar or broth assays.

In addition to microbial metabolites, whey-derived antimicrobial peptides have the potential to contribute to the bioactivity of fermented whey. Lactoferricin and other whey-derived antimicrobial peptides have been described as bioactive sequences capable of disrupting microbial membranes through cationic interactions and pore formation (155). Bovine lactoferrin may also sequester iron from bacterial pathogens, whereas digestion-derived peptides, such as lactoferricin and lactoferrampin, possess antibacterial and antifungal properties (28).

Antibiofilm activity is another important dimension of antimicrobial potential. Biofilms increase microbial resistance to environmental stress and antimicrobials and are highly relevant in food processing, oral health and gastrointestinal pathogen persistence. Fermented whey showed strong inhibition of *S. mutans* biofilm formation (118), while broader postbiotic studies have described biosurfactants, bacteriocins, organic acids, and teichoic acids as compounds capable of reducing pathogen adhesion or disrupting biofilm formation (154, 159). EPS from *Lactiplantibacillus plantarum* postbiotics has also been shown to reduce aggregation, co-aggregation, and hydrophobicity in oral biofilm-associated bacteria (162). Although this evidence is not entirely whey-specific, it supports the relevance of EPS-mediated interference with adhesion and biofilm formation as a possible mechanism in fermented whey preparations produced using EPS-forming strains.

### 7.3 Anti-inflammatory effects

Fermented whey-derived postbiotic-associated preparations can exert anti-inflammatory and immunomodulatory effects through several complementary mechanisms, including SCFAs, whey-derived BAPs, EPSs, soluble microbial metabolites, and inactivated microbial cells or cell wall fragments in some preparations (23, 24, 98–100, 154). However, the evidence should be evaluated carefully because only some studies provide direct data on whey-based preparations, while others offer broader mechanistic support from general postbiotic or LAB-derived preparations (7, 24, 28, 155).

The anti-inflammatory interpretation is strengthened by a systematic review which evaluated 39 studies on postbiotic formulations and inflammation (24). The review concluded that postbiotics may exert multifaceted anti-inflammatory effects by modulating cytokine expression, immune cell signaling pathways, epithelial barrier integrity, and Th1/Th2 and Treg/Th17 balance. This provides a stronger evidence base than narrative reviews alone, although the authors also emphasized the study heterogeneity, methodological limitations, and risk of bias.

A major mechanism linking postbiotics with anti-inflammatory activity is the production or delivery of SCFAs. They can modulate inflammation through G-protein-coupled receptors, epithelial barrier maintenance, regulatory T-cell differentiation, and cytokine regulation. Butyrate is particularly important because it serves as an energy source for colonocytes and is associated with anti-inflammatory and barrier-supporting effects, whereas propionate and acetate contribute to metabolic and immune regulation (98–100).

Whey-derived peptides have the potential to contribute to anti-inflammatory and immunomodulatory activity. An interesting study described whey-derived peptides as capable of modulating inflammatory pathways by reducing pro-inflammatory mediators such as TNF-α, IL-6, and IL-1β, increasing anti-inflammatory IL-10, and influencing NF-κB-, PPARγ-, and TLR-related signaling (155). Additional whey-specific support is described in a paper summarizing studies in which fermented whey or whey-protein products were linked to reduced colitis severity, decreased pro-inflammatory mediators such as C-reactive protein, IL-4, and TNF-α, and increased anti-inflammatory or barrier-related responses such as TGF-β and intestinal IgA (7).

EPSs and microbial structural components may provide additional immunomodulatory effects. LAB-derived EPSs can influence intestinal barrier function and inflammatory cytokine production; for example, EPS from Lactiplantibacillus plantarum has been reported to upregulate tight-junction proteins such as ZO-1 and occludin, repress claudin-2, and reduce pro-inflammatory cytokines, including IFN-γ, IL-6, and TNF-α (98). Inactivated microbial cells and cell-wall fragments, including peptidoglycan, lipoteichoic acids, teichoic acids, surface proteins, and bacterial DNA, may also interact with host pattern-recognition receptors and influence innate and adaptive immune responses (23, 99, 154, 159).

Experimental non-whey studies further support the anti-inflammatory potential of microbial postbiotic fractions, including reduced inflammatory cytokines, improved intestinal morphology, increased SCFAs and enhanced tight-junction protein expression in animal or cellular models (31, 157). Disease-focused reviews also connect postbiotics with cytokine regulation, dysbiosis, impaired barrier function and low-grade inflammation, although these sources do not provide direct evidence for fermented whey products (100, 163).

Generally, the anti-inflammatory activity of fermented whey should not be viewed solely as a change in cytokine levels. It can also involve reduced oxidative stress, improved epithelial barrier integrity, increased mucus production, enhanced tight-junction expression, reduced pathogen adhesion, improved microbiota composition and SCFA-mediated epithelial signalling. Nevertheless, direct fermented whey studies using immune cell models, intestinal epithelial barrier assays, animal models, and human interventions remain necessary before firm anti-inflammatory health claims can be made.

### 7.4 Effects on gastrointestinal system

The gastrointestinal system is one of the most relevant target sites for fermented whey-derived postbiotic preparations because it is directly exposed to whey-derived peptides, organic acids, SCFAs, EPSs, microbial metabolites, and, in some products, inactivated microbial cells or cell wall components. Potential gastrointestinal effects may include modulation of gut microbiota, support of epithelial barrier integrity, production or delivery of SCFAs, reduction of intestinal permeability, inhibition of gastrointestinal pathogens, support of mucosal immune balance, and effects on gut-derived metabolic signaling (7, 28, 98–100, 118, 155), as illustrated in Figure 3.

**Figure 3:**
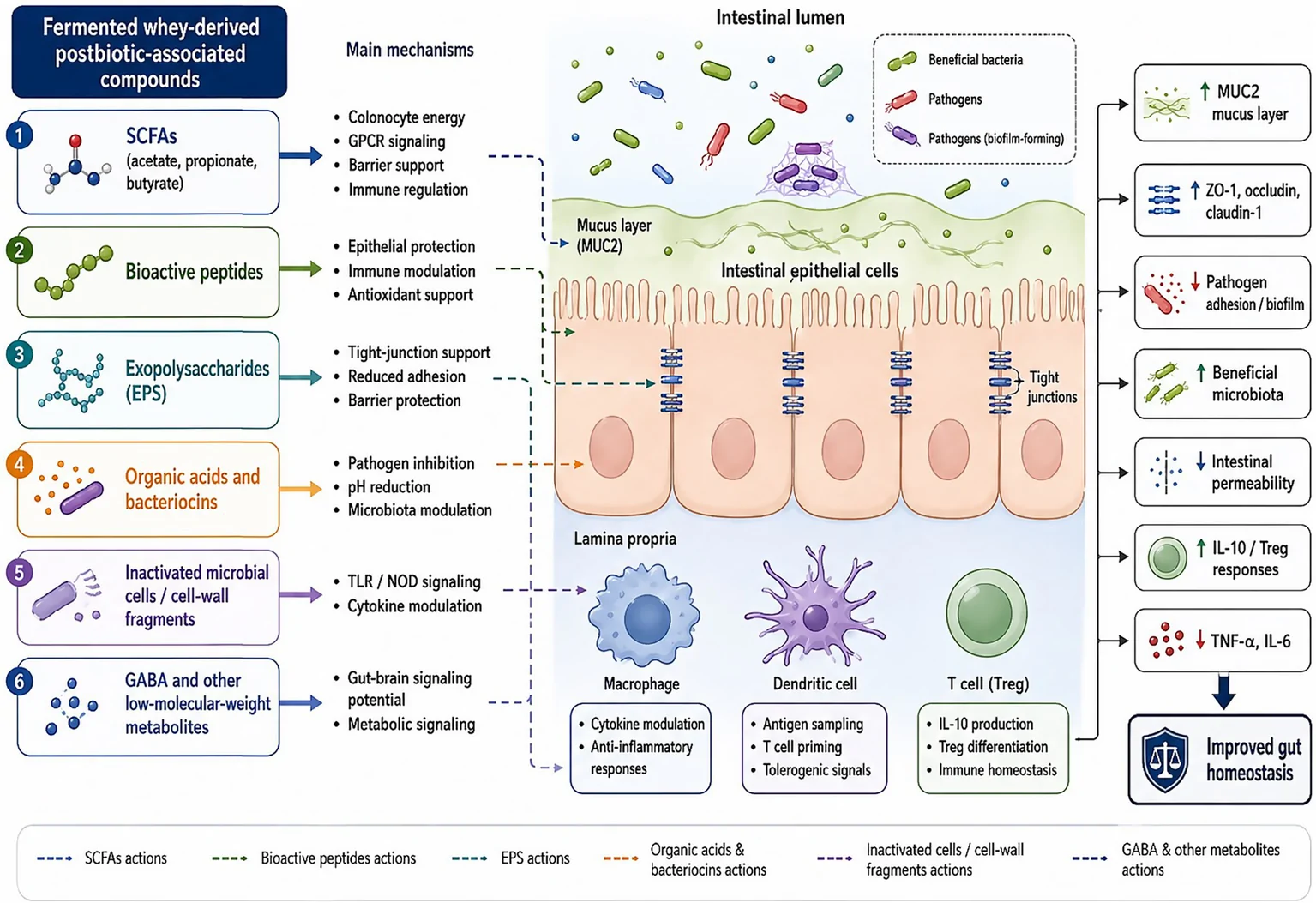
Possible gastrointestinal mechanisms of fermented whey-derived postbiotics [Created with ChatGPT (OpenAI, GPT-5.5) and edited by the authors]. The schematic figure does not reproduce copyrighted graphical elements.

Whey-focused synthesis supports this interpretation by linking fermented whey and whey protein products with intestinal immunity, gut microbiota modulation, SCFA production, villus morphology, goblet cells, intraepithelial lymphocytes, and barrier-related responses (7). This positions fermented whey not only as a source of bioactive compounds, but also as a food matrix capable of influencing gut-associated structure and function.

Another study added a relevant gastrointestinal safety dimension by showing the partial degradation of β-lactoglobulin, the major whey allergen, during LAB fermentation of whey, with strain-dependent reductions of approximately 12–33% (35).

Whey-derived bioactive peptides may contribute to gastrointestinal effects by acting locally in the intestinal lumen and epithelium, where they may influence mucosal immune responses, macrophage activity, cytokine balance and epithelial protection (155). In addition to these functional effects, LAB fermentation of whey may improve the gastrointestinal suitability of whey-based products by partially degrading β-lactoglobulin, the major whey allergen, with reported strain-dependent reductions of approximately 12–33% (35).

Further support for the gastrointestinal relevance of fermented whey comes from studies showing that fermented whey preparations influenced gut immunity and inflammation in experimental models (28). A second major gastrointestinal mechanism is epithelial barrier protection. Disruption of tight junctions and increased permeability are associated with gastrointestinal disorders, such as inflammatory bowel disease, irritable bowel syndrome, foodborne infections, and metabolic endotoxaemia. ZO-1 and occludin are key tight-junction proteins involved in maintaining intestinal barrier integrity (98). In addition, postbiotic components, such as SCFAs, bacteriocins, lipoteichoic acids, surface-layer proteins, and secreted proteins, can contribute to barrier function through their effects on MUC2, ZO-1, occludin, claudin-1, and claudin-4 (99). Butyrate is also relevant to this barrier-focused interpretation because it serves as an energy source for enterocytes and regulates gene expression via histone deacetylase inhibition (23).

Microbiota modulation is another important gastrointestinal effect. Postbiotic preparations influence the gut microbiota directly through antimicrobial compounds and indirectly through organic acids, SCFAs, pH reduction and substrate cross-feeding. This process involves pathogen inhibition by compounds such as bacteriocins, environmental modification through organic acids, and adhesion-related effects mediated by structural components (99). In fermented whey, these mechanisms can be relevant because fermentation can generate organic acids, antimicrobial peptides, bacteriocin-like compounds, EPS and other metabolites capable of influencing microbial ecology.

Other gut-relevant effects may include GABA production, lactose hydrolysis, and reduction of undesirable nitrogenous compounds. LAB-fermented whey has been reported to produce GABA and reduce the levels of selected biogenic amines, including putrescine and cadaverine (118). Some LAB used in fermented dairy and whey systems also produce β-galactosidase, which contribute to lactose hydrolysis and improved lactose digestibility.

Broader disease-oriented reviews support the relevance of microbiota modulation, SCFA-mediated signalling, barrier protection and cytokine regulation, but they should not be interpreted as direct evidence for fermented whey in gastrointestinal disorders (100, 163, 164).

Overall, fermented whey-derived postbiotic-associated preparations may influence gastrointestinal function through several interconnected mechanisms, including SCFA production, epithelial barrier support, mucosal immune modulation, pathogen inhibition, microbiota modulation, peptide generation, and improvement of product digestibility or tolerability (7, 23, 28, 98–100, 118). However, most available evidence is still derived from chemical profiling, fermentation outputs, in vitro assays, or preclinical models, and therefore remains insufficient to support specific gastrointestinal health claims (24, 28, 35, 118). Future studies should therefore include simulated gastrointestinal digestion, gut microbiota fermentation models, epithelial barrier assays, cytokine profiling, animal models, and well-designed human intervention trials before specific gastrointestinal health claims can be made (24, 40, 84, 155).

### 7.5 Potential health benefits and functional applications

Ideally, fermented whey-derived postbiotic-associated preparations will provide a multifunctional platform in which nutritional valorization, bioactive compound generation, food preservation, and potential health-promoting effects converge. The functional application potential of fermented whey is supported by studies demonstrating that whey and whey proteins can serve as preservatives, high-protein dietary ingredients, and sources of physiologically active substances in fermented foods (7). This is particularly relevant for connecting the bioactivities discussed in Sections 7.1–7.4 with practical applications in fermented beverages, dairy products, bakery products, meat products, whey vinegar, and other functional food systems (7, 28, 30, 34, 165–169).

The evidence reviewed in the previous subsections suggests that fermented whey can act both as a substrate for microbial transformation and as a source of whey-protein-derived bioactive peptides. Through fermentation, whey is enriched with organic acids, SCFAs, antimicrobial peptides, bacteriocin-like substances, EPSs, phenolic-associated compounds, GABA, ACE-inhibitory peptides, antioxidant peptides and immunomodulatory components (3, 7, 28, 35, 70, 118, 155). These compounds can contribute to antioxidative, antimicrobial, anti-inflammatory and gastrointestinal effects, although the strength of evidence differs between compound classes and biological endpoints (7, 28, 35, 98, 99, 118, 155)

From a health perspective, the most plausible benefits of fermented whey postbiotic-associated preparations are related to oxidative balance, pathogen control, gut barrier protection, mucosal immune regulation, SCFA-mediated gut effects, and metabolic or cardiovascular support (24, 28, 34, 98–100, 118, 167, 168, 170, 171). Fermented whey may also have potential as a gut-supporting ingredient. SCFA production, lactate and acetate generation, reduction of selected biogenic amines, production of GABA, and release of bioactive peptides improve the functional profile of whey-based products (3, 35, 118, 155, 169). Condensed preclinical data indicate that fermented whey products can affect intestinal immune responses and inflammation, including colitis-related models (24, 28, 34, 46, 167, 168).

The antimicrobial properties of fermented whey-derived postbiotic fractions have important food safety applications. Fermented whey may inhibit foodborne and spoilage microorganisms through acidification, bacteriocins, bacteriocin-like substances, antimicrobial peptides, organic acids and other metabolites (28, 98, 99, 118, 154, 157, 160). In practical food systems, fermented whey-derived preparations can be used as natural preservatives, bioactive ingredients, functional beverage bases, spray-dried powders, starter-derived fermentates, or active packaging components (28, 84, 171–174).

Industrial adoption will require more than just the demonstration of antioxidant or antimicrobial activity. Key barriers, such as strain specificity, substrate effects, analytical standardization, quantification, formulation stability, regulatory alignment, and reproducible functionality across food systems, still require attention (84, 152, 174). These issues are particularly important for fermented whey because changes in whey type, protein concentration, lactose level, heat treatment, and supplementation can strongly alter the final bioactive profile (3, 28, 30, 35, 70). Future research should therefore combine detailed chemical characterization with metabolomic, peptidomic and SCFA profiling, stability testing, food-matrix validation, simulated digestion, gut models, safety assessment and, ultimately, well-designed human intervention studies (3, 28, 30, 35, 70).

## 8 Whey-based functional beverages: fermented, probiotic, synbiotic and postbiotic systems

### 8.1 Development and classification of whey-based functional beverages

Whey is a suitable substrate for the development of a broad range of fermented functional beverages because of its lactose, protein, mineral, and vitamin content (6, 165). Fermentation with lactic acid bacteria (LAB), kefir-associated microbial consortia, and yeasts can improve the sensory, technological, and functional properties of whey-based beverages through acidification, microbial metabolism, proteolysis, and the formation of fermentation-derived bioactive compounds (63, 153). However, fermented whey beverages should not automatically be classified as postbiotic products. Their appropriate classification depends on the microbial viability and composition of the final product. Whey beverages containing viable beneficial microorganisms are more appropriately described as probiotic or fermented whey beverages, whereas products containing both probiotics and prebiotic substrates may be classified as synbiotic beverages. In contrast, a defined postbiotic whey beverage should contain a preparation of inanimate microorganisms and/or their components that meets the accepted definition of a postbiotic preparation. Fermented whey containing mainly soluble microbial metabolites but lacking characterized inactivated microbial biomass should instead be described as a fermented whey beverage or a metabolite-enriched fermented whey beverage. Recent interest has increasingly shifted toward postbiotic-enriched beverages owing to the greater stability and safety of postbiotics compared with live probiotic cultures (46, 99). Plant-derived ingredients such as citrus pomace, grape seed flour, fruit extracts, and soluble fibers may further enhance the nutritional, antioxidant, sensory, or technological properties of whey beverages (34, 167, 168). Their contribution, however, should be distinguished from bioactive compounds generated through microbial fermentation because plant-derived polyphenols and fibers are not postbiotic components. Accordingly, whey-based functional beverages discussed in this review are classified according to their processing and biological composition rather than being collectively designated as postbiotic beverages.

Consequently, whey-based functional beverages, including appropriately defined postbiotic formulations, represent a promising category of sustainable functional foods that combine dairy by-product valorization with advanced fermentation biotechnologies.

### 8.2 Fermented, probiotic, synbiotic, and postbiotic whey beverages: recent research and applications

Recent research has explored a wide range of whey-based functional beverages, including conventional fermented, probiotic, synbiotic, plant-enriched, yeast-fermented, and postbiotic-oriented systems. These categories should be differentiated according to the presence or absence of viable microorganisms, use of prebiotic substrates, presence of characterized inactivated microbial components, and origin of the bioactive compounds detected in the final beverage (Figure 4).

**Figure 4:**
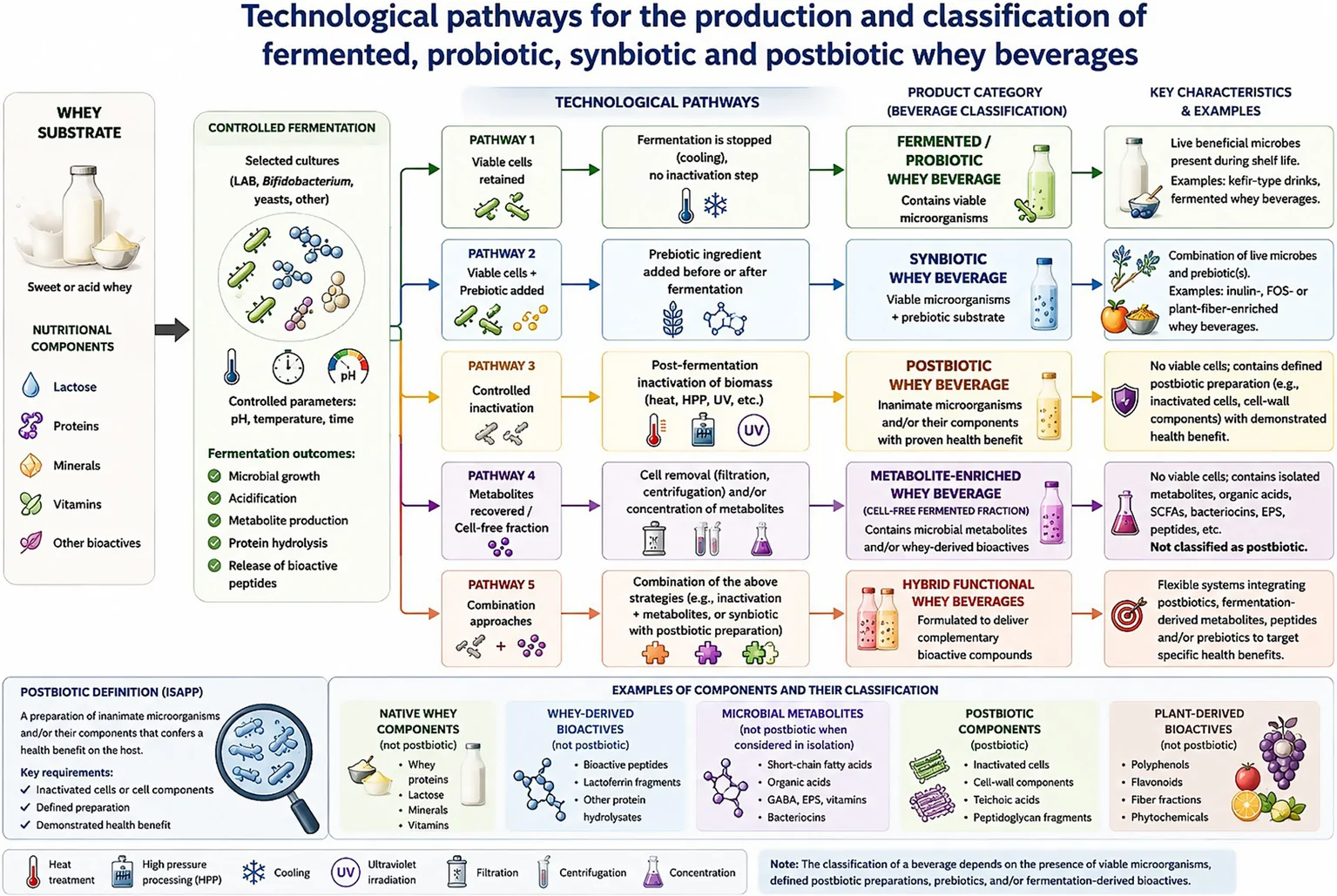
Technological pathways for the production and classification of fermented, probiotic, synbiotic and postbiotic whey beverages [Created with ChatGPT (OpenAI, GPT-5.5) and edited by the authors]. The schematic figure does not reproduce copyrighted graphical elements.

Kefir-derived whey beverages represent fermented microbial systems containing complex associations of lactic acid bacteria and yeasts. Studies have shown that whey beverages fermented with kefir grains may exhibit improved physicochemical characteristics, probiotic viability, and sensory acceptability during refrigerated storage (165). Because viable microorganisms are retained in these systems, they should primarily be regarded as fermented or probiotic whey beverages rather than postbiotic beverages.

Plant-enriched whey fermentation systems have also attracted considerable interest. Whey fermented with *Lentilactobacillus kefiri* DH5 and supplemented with citrus pomace extract has been associated with metabolic and gut microbiota-related effects in an experimental model reported in a previous study (167). However, these effects should not be attributed solely to microbial fermentation because citrus pomace provides polyphenols and dietary fiber that may independently or synergistically contribute to the observed response.

Similarly, grape seed flour supplementation increased the bioactive potential of fermented whey systems, particularly in terms of antioxidant and anti-inflammatory activities (34, 168). In these formulations, the functional response should be interpreted as resulting from interactions among whey constituents, fermentation-derived metabolites, microbial activity, and plant-derived polyphenols, rather than from postbiotics alone. Beyond antioxidant applications, recent studies have reported the targeted physiological effects of whey-derived postbiotic systems, including anti-sarcopenic activity, glycemic control, metabolic regulation, and immune modulation (34). Whey-based beverages containing deliberately inactivated microbial cells are particularly relevant to the postbiotic concept. For example, a beverage containing inactivated *Lacticaseibacillus casei* has been reported to influence postprandial glycemic responses in healthy adults. Because the microbial cells are inactivated, this type of formulation is conceptually distinct from conventional probiotic or fermented whey beverages and may be considered within a postbiotic framework, provided that the final preparation fulfills the accepted postbiotic definition.

Synbiotic whey beverages represent a separate functional beverage category because they combine viable probiotic microorganisms with prebiotic substrates. Improved probiotic survival and gastrointestinal functionality have been reported in whey beverages containing *Lactobacillus acidophilus* and inulin (175). Because their intended functionality depends on viable microbial cells, these products should not be classified as postbiotic beverages. Increased SCFA formation reported in fiber-supplemented whey fermentation systems should also be interpreted in relation to the specific substrate and microbial system rather than generalized to conventional whey fermentation (3).

Kombucha-type and yeast-fermented whey beverages are additional fermentation-based product categories. Kombucha-inspired whey beverages enriched with tea extracts have demonstrated increased antioxidant capacity and acceptable sensory characteristics (176), while fermentation with *Kluyveromyces marxianus* and *Saccharomyces cerevisiae* has been investigated primarily to improve fermentation efficiency and aroma development (4). These systems should be described according to their microbial composition and processing characteristics, rather than being automatically classified as postbiotic beverages.

As summarized in Table 6, whey-based functional beverages comprise heterogeneous technological and biological systems and should not be considered a single postbiotic product category. Kefir-associated and probiotic beverages contain viable microorganisms, and synbiotic systems additionally include prebiotic substrates, whereas plant- and tea-enriched beverages derive part of their functional properties from non-microbial bioactive ingredients. Only formulations containing a defined preparation of inactivated microorganisms and/or their components that demonstrate an associated health benefit should be classified as postbiotic beverages.

**Table 6:** Selected whey-based functional beverages and their properties.

Beverage system	Microorganisms	Added functional ingredients	Main bioactive effects	Reference
Whey + citrus pomace extract	*Lentilactobacillus kefiri* DH5	Citrus polyphenols	Anti-obesity, gut microbiota modulation	(167)
Whey + grape seed flour extract	*L. kefiri* DH5	Grape polyphenols	Anti-inflammatory, anti-sarcopenic	(34)
Whey + inulin	*L. plantarum*, *B. lactis*	Soluble fibers	SCFA production, antioxidant activity	(3)
Fermented whey kefir beverages	Kefir grains	Whey protein concentrate	Improved probiotic viability and sensory quality	(165)
Synbiotic whey drinks	*Lactobacillus acidophilus*	Inulin	Enhanced probiotic survival and acceptability	(185)
Fruit-fortified whey beverages	LAB cultures	Fruit juices and extracts	Improved antioxidant capacity and sensory properties	(181)
Kombucha-type whey beverages	Mixed cultures	Tea extracts and plant bioactives	Enhanced antioxidant activity	(176)
Yeast-fermented acid whey beverages	*Kluyveromyces marxianus*, *Saccharomyces cerevisiae*	Acid whey	Improved aroma profile and fermentation efficiency	(4)

### 8.3 Technological production of fermented and postbiotic whey beverages

The technological production of whey-based functional beverages depends on the intended product. Whey pretreatment may include pasteurization, ultrafiltration, concentration, and deacidification, particularly in the case of acid whey (177–179). These operations contribute to microbiological safety and may improve the nutritional, sensory, and technological suitability of whey for fermentation.

Controlled fermentation represents the central biotechnological stage during which selected LAB, yeasts, or mixed cultures metabolize whey constituents, produce microbial biomass, and generate extracellular metabolites. Microbial proteolysis may simultaneously release whey-derived bioactive peptides from the protein fraction. However, at this stage the product should be regarded as fermented whey rather than automatically as a postbiotic preparation.

The downstream process depends on the intended beverage product. If viable microorganisms are retained, the product may be classified as fermented or probiotic beverage. If viable microorganisms are combined with a prebiotic substrate, the system may be classified as a synbiotic. In contrast, when a postbiotic beverage is intended, fermentation should be followed by a defined inactivation step, after which the absence of viable cells and the composition and biological activity of the resulting preparation should be characterized.

Depending on the intended formulation, subsequent operations may include concentration, spray drying, freeze drying, microencapsulation, or other stabilization technologies aimed at preserving fermentation-derived bioactive compounds and facilitating their incorporation into the final beverage matrix (1, 30, 46). Emerging technologies, including high-pressure processing and pulsed electric fields, may also be considered, although their effects on microbial inactivation, compound stability, and final product classification must be evaluated for each system.

### 8.4 Nutritional and functional properties of whey-based functional beverages

Whey proteins possess a high biological value and are particularly rich in branched-chain amino acids (BCAAs), including leucine, isoleucine, and valine, which are associated with muscle protein synthesis, tissue recovery, metabolic regulation, and prevention of sarcopenia (180). Consequently, whey-derived beverages are increasingly being investigated for their applications in sports nutrition and clinical dietary formulations.

Fermentation may further enhance the functional value of whey beverages through microbial metabolism and proteolytic transformation of whey constituents (46). The proteolytic activity of LAB can improve protein digestibility and release whey-derived bioactive peptides. These peptides should be distinguished from postbiotic components because they originate from the whey protein substrate.

Antioxidant capacity is among the most frequently investigated functional properties of fermented whey beverages. This may result from the combined contribution of whey-derived peptides, microbial metabolites, and plant-derived polyphenols when botanical ingredients are incorporated into the formulation (168, 181). Polyphenol-rich ingredients. such as citrus pomace and grape seed flour, may increase the chemical antioxidant capacity of the beverage (167, 168). However, results obtained from chemical antioxidant assays should be interpreted as evidence of radical-scavenging or antioxidant capacity and not as direct evidence of systemic antioxidant effects in humans.

Fermentation-derived SCFAs, EPS, and microbial cell-associated components have been associated with gut-related biological effects in different experimental systems (2, 3). However, these components should be interpreted separately according to their origin and evidence level. SCFAs detected in the fermented beverage should be distinguished from those subsequently generated by the intestinal microbiota after consumption, while microbial cell components are relevant to a postbiotic designation only when they are present within a defined inactivated microbial preparation.

However, despite the promising biological functionality of whey-based functional beverages and defined postbiotic formulations, several limitations remain insufficiently investigated, including the bioavailability of fermentation-derived metabolites and postbiotic-associated components, the interactions between different bioactive compounds, and their stability during industrial processing and storage.

As summarized in Table 7, whey-based functional beverages contain a heterogeneous mixture of nutritional and bioactive components originating from the native whey matrix, microbial fermentation, proteolytic transformation of whey proteins, and, where applicable, added plant ingredients. These components should not be collectively classified as postbiotics. Only defined preparations containing inactivated microorganisms and/or their components that confer demonstrated health benefits meet the accepted postbiotic definition. Consequently, the biological functionality of whey-based beverages should be interpreted according to the origin of the active components and the level of supporting evidence.

**Table 7:** Major functional compounds in whey-based functional beverages and their physiological effects.

Functional compound	Main physiological effect	References
Whey proteins	Muscle protein synthesis and recovery	(2, 180)
Bioactive peptides	Antihypertensive and antioxidant activity	(38, 145)
Short-chain fatty acids (SCFAs)	Gut microbiota modulation	(3, 167)
Exopolysaccharides (EPS)	Immunomodulatory activity	(46, 172)
Organic acids	Antimicrobial effects	(28, 190)
Polyphenols	Antioxidant and anti-inflammatory activity	(168, 181)
Bacteriocins	Natural preservation	(28, 166)
Vitamins and minerals	Nutritional support and metabolic regulation	(6, 180)

### 8.5 Challenges and future perspectives for whey-based functional and postbiotic beverages

Despite considerable progress achieved in developing of whey-based functional beverages and emerging defined postbiotic formulations, challenges related to metabolite stability during processing and storage, as well as physicochemical changes such as phase separation, sedimentation, and excessive acidity, continue to limit product quality and consumer acceptability (1).

Another important limitation is the variability of fermentation-derived bioactive profiles and, where applicable, postbiotic preparation composition associated with differences in microbial strains, fermentation conditions, substrate composition, and processing technologies, which complicate industrial standardization and reproducibility between production batches.

Furthermore, despite promising in vitro and experimental results, the clinical validation of whey-based postbiotic beverages remains limited, and additional human studies are required to confirm their long-term physiological effects and health claims (169).

Future research should focus on precision fermentation technologies to improve metabolite production, process control, and product consistency. Advanced approaches, including AI-assisted process optimization, real-time fermentation monitoring, and predictive microbial modeling, may improve industrial scalability and product standardization. The growing demand for sustainable clean-label products is also expected to support the development of naturally stabilized whey-based postbiotic beverages.

Emerging trends in sustainable dairy biotechnology emphasize circular bioeconomy principles, dairy side-stream valorization, and the incorporation of plant-derived bioactives into whey fermentation systems, supporting the development of more stable and functional whey-based postbiotic beverages.

### 8.6 Safety, regulatory and standardization considerations for whey-based functional beverages

Regulatory and safety requirements strongly depend on the category of the final whey-based beverage. Products containing viable microorganisms require strain identification, viability control, microbiological safety assessment, and appropriate labeling og the products. Defined postbiotic beverages additionally require documentation of the microbial source, inactivation procedure, absence of viable cells where applicable, compositional characterization, safety, and evidence supporting the claimed biological effects. Fermentation-derived metabolites, purified bacteriocins, and whey-derived peptides should be assessed according to their specific regulatory status rather than being automatically classified as postbiotics.

The increasing commercialization of postbiotic products has highlighted the need for clear regulatory frameworks governing their safety, characterization, labeling, and health claims. Compared with live probiotics, postbiotics offer greater stability during processing and storage while reducing the microbiological risks associated with viable microorganisms (3, 20).

However, harmonized international regulations covering postbiotic characterization, dosage standardization, labeling, and health claims remain limited (46). In the European Union, the European Food Safety Authority (EFSA) applies strict scientific criteria for the approval of health claims related to microbial-derived products, requiring robust evidence of safety, efficacy, and reproducibility (46). Consequently, variability in microbial strains, fermentation conditions, and metabolite composition continues to complicate the regulatory approval and industrial standardization of postbiotic-enriched functional beverages (1, 30).

Postbiotics are consistent with the growing demand for sustainable clean-label food products because they may reduce the need for synthetic preservatives while improving product stability. Consequently, whey-based postbiotic beverages represent a promising category of functional foods with considerable commercial potential, provided that appropriate regulatory requirements are met.

## 9 Active food packaging based on whey-derived postbiotic preparations and fermentation-derived bioactive compounds

### 9.1 Concept of active functional packaging

The transition from traditional passive barriers to active packaging represents a fundamental shift in food science, moving toward systems that dynamically interact with the food product or its internal environment to maintain quality and safety (182, 183). While the primary goal of active packaging is to extend shelf life through the controlled release of antimicrobial or antioxidant agents, functional packaging further enables the delivery of bioactive compounds with potential health benefits (173, 184).

Among the various food-packaging materials, plastics are widely used owing to their lightweight, versatility, and cost-effectiveness. Moreover, they can be easily tailored to provide specific barrier, thermal, and/or mechanical properties. Moreover, in recent decades, awareness of the environmental impact of fossil-based plastics has increased. Therefore, conventional plastics, such as polyethylene (PE), polypropylene (PP), polyethylene terephthalate (PET), polyvinyl chloride (PVC), and polystyrene (PS), are increasingly being replaced by more sustainable bio-based and biodegradable alternatives. Examples include materials based on polysaccharides, proteins, lipids, and their combinations (composites) (185–188). The properties of the selected polymer matrix affect the release behavior of active inclusions (antimicrobials, antioxidants) and include mixture composition and component proportions, polymer molecular weight, crystallinity, hydrophilicity, cross-linking, and porosity. Therefore, the release mechanism of active compounds from the packaging materials is governed by the physical and chemical processes that transport an active agent from the packaging matrix to the target site-the food surface or headspace in the packaging system (187, 189).

Compared with live probiotics, whey-derived postbiotic preparations offer greater thermal and storage stability, making them particularly suitable for incorporation into food-packaging materials processed uisng techniques such as extrusion and solvent casting (190, 191). The use of whey as a substrate for the production of postbiotic preparations also supports circular bioeconomy strategies by valorizing a major dairy by-product (3, 10). The characteristics of the fermentation substrate significantly influence the composition of postbiotic preparations, the profile of fermentation-derived bioactive compounds, and their biological activity. Therefore, careful substrate selection and standardization are essential for producing whey-derived postbiotic preparations with consistent bioactivity and predictable release of active compounds in food packaging applications (3, 30).

### 9.2 Antimicrobial functionalities

Whey-derived postbiotic preparations are increasingly being investigated for application in active packaging systems because of their potential to provide antimicrobial protection and extend the shelf life of various food products (174, 192). Their antimicrobial activity is primarily attributed to the presence of fermentation-derived bioactive compounds, including bacteriocins, organic acids, and antimicrobial peptides, which interfere with essential microbial processes and suppress the growth of foodborne pathogens (172, 173, 193). The high stability of these bioactive compounds supports the incorporation of whey-derived postbiotic preparations into active packaging materials (190, 194).

Among the antimicrobial bioactive compounds present in whey-derived postbiotic preparations, bacteriocins have been widely investigated for application in active packaging because of their high efficacy against foodborne pathogens (190, 195).

Nisin is one of the most extensively investigated bacteriocins for packaging applications, particularly against Gram-positive bacteria such as *Listeria monocytogenes* and *Staphylococcus aureus* (196, 197). By binding to lipid II, nisin disrupts cell wall synthesis and membrane integrity, leading to leakage of intracellular components and cell death (194, 198, 199). Organic acids, primarily lactic and acetic acids, further contribute to the antimicrobial activity of whey-derived postbiotic preparations as fermentation-derived metabolites by lowering environmental pH and disturbing intracellular homeostasis, thereby suppressing the growth of spoilage and pathogenic microorganisms (200, 201).

Antimicrobial peptides (AMPs) derived from natural sources exhibit broad-spectrum antimicrobial activity by destabilizing microbial membranes and, in some cases, interfering with intracellular targets involved in DNA, RNA, and protein synthesis (202–204). Owing to their multiple mechanisms of action and high stability, AMPs are promising candidates for incorporation into active food packaging materials (205, 206).

The antimicrobial activity of packaging systems is based on several mechanisms, including cell membrane disruption, interference with cell wall and protein synthesis, inhibition of enzyme activity, and induction of oxidative stress (201, 207, 208) (Figure 5). Beyond their direct antimicrobial effects, whey-derived postbiotic preparations also inhibit biofilm formation by disrupting quorum sensing, suppressing bacterial motility, and promoting the degradation of the extracellular matrix (193, 194, 209).

**Figure 5:**
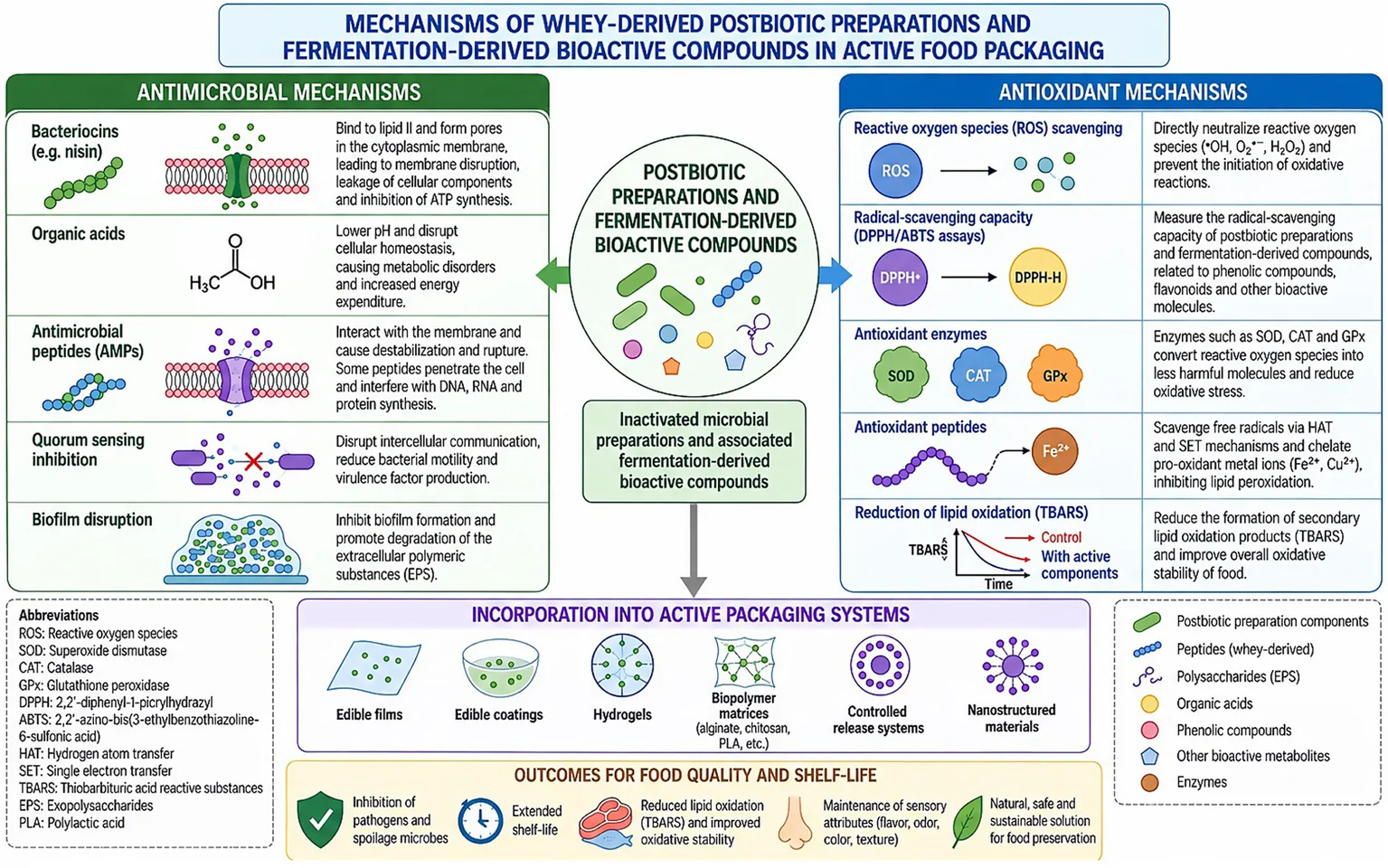
Proposed antimicrobial and antioxidant mechanisms of whey-derived postbiotic preparations and fermentation-derived bioactive compounds in active food packaging systems [Created with ChatGPT (OpenAI, GPT-5.5) and edited by the authors]. The schematic figure does not reproduce copyrighted graphical elements.

The incorporation of whey-derived postbiotic preparations into biopolymer matrices, including chitosan, alginate, and polylactic acid (PLA), enables the controlled release of fermentation-derived bioactive compounds (210–212). Furthermore, combining whey-derived postbiotic preparations with nanostructured delivery systems further improves their stability and antimicrobial performance, offering an effective strategy for controlling microbial contamination during food processing and storage (213).

### 9.3 Antioxidant functionalities

In addition to their antimicrobial role, whey-derived postbiotic preparations possess significant antioxidant potential, which contributes to the preservation of food quality and stability (190). This activity is associated with postbiotic-associated components and fermentation-derived bioactive compounds, including antioxidant peptides, exopolysaccharides (EPS), phenolic compounds, and antioxidant enzymes, which collectively reduce oxidative deterioration and improve the functional performance of active packaging systems (174, 194). When incorporated into packaging materials, these compounds suppress lipid oxidation, thereby preserving the sensory characteristics and nutritional quality of food during storage (212, 214).

The antioxidant potential of postbiotic preparations and fermentation-derived bioactive compounds can be evaluated using assays such as DPPH and ABTS, which assess their radical-scavenging activities (193, 211). This protective effect is mainly attributed to bioactive compounds associated with fermentation, including phenolic compounds, flavonoids, and antioxidant peptides, which may contribute to the oxidative stability of active packaging systems (174, 212).

Antioxidant peptides, along with other antioxidant bioactive compounds, contribute to oxidative stability by neutralizing reactive oxygen species and limiting lipid peroxidation. In addition, antioxidant peptides can chelate pro-oxidative metal ions, thereby further reducing oxidative deterioration and helping preserve food quality (190, 215).

The incorporation of postbiotic preparations into biopolymer matrices further enhances oxidative stability by reducing TBARS values and limiting lipid oxidation in meat and fish products (212, 216).

The combined effect of antioxidant bioactive compounds and oxygen-barrier properties of these packaging materials (217) delays the development of rancidity, off-flavors, and off-odors, thereby extending shelf life while preserving the sensory quality of food products (174). In most cases, the effectiveness of active antioxidant packaging depends on the balance between the release rate of active compounds and the kinetics of food oxidation; therefore, determining the release kinetics of antioxidants from packaging materials is essential for designing effective active packaging systems (189).

### 9.4 Incorporation of postbiotic preparations into biopolymer films and coatings

The integration of whey-derived postbiotic preparations into biopolymer matrices, including alginate, chitosan, protein-based materials, polylactic acid (PLA), polyhydroxyalkanoates (PHA), and others, represents an important strategy for developing functional active packaging systems (173, 190). Evidence from related active-packaging systems has further demonstrated that starch-based biodegradable films reinforced with basil essential oil, carboxymethyl cellulose, and citric acid exhibit enhanced mechanical performance and functional properties, highlighting their potential as sustainable materials for advanced active food packaging (189, 218, 219).

The choice of application technique depends on the packaging format of the product. Industrial processes such as extrusion and solvent casting are primarly used for films production, whereas edible coatings are typically applied by dipping, spraying, or brushing directly onto the food surface (220–222). Plasticizers, particularly glycerol and sorbitol, play an essential role in improving the flexibility of biopolymer matrices by reducing their intermolecular interactions and preventing brittleness (223). Studies on related biopolymer systems indicate that hydrogen bonding and electrostatic interactions between incorporated bioactive components and the polymer matrix can contribute to matrix stability (224). These interactions may also influence the retention and controlled release of bioactive compounds incorporated into active packaging systems (187, 189).

Whey protein isolate (WPI) and whey protein concentrate (WPC) have attracted considerable attention as oxygen-barrier materials and matrices for incorporating bioactive compounds into food packaging systems. WPI and WPC also contribute to whey valorization and support circular bioeconomy strategies by enabling sustainable utilization of dairy by-products (225).

In general, the food-packaging system refers to the direct contact between food-contact materials (packaging) and food. The main migration processes of antioxidant and antimicrobial compounds include (i) diffusion within the polymer material, (ii) transfer from the packaging material to the food surface, and (iii) distribution across interfaces. However, the release of active compounds from packaging materials is rarely controlled by a single process; in the package–headspace–food system, gas-phase diffusion is one of the major migration phenomena affecting the equilibrium distribution of active compounds among packaging materials, headspace, and food. Moreover, diffusion mainly depends on the polymer type, polymer swelling, matrix erosion or degradation, partitioning into heterogeneous foods, and, more rarely, osmotic effects. In addition, stimuli-responsive release mechanisms may occur, with triggering factors including temperature, pH, moisture, light, and the presence of microorganisms. Therefore, a good understanding of these processes is essential for the proper design of packaging materials because the same formulation can exhibit substantially different release kinetics depending on the characteristics of the packaged food, including its composition (e.g., fat/protein ratio), pH, and water activity (187–189, 226, 227).

### 9.5 Application of postbiotic preparations for shelf-life extension

The primary objective of active packaging incorporating postbiotic preparations is to delay microbial and oxidative spoilage, thereby improving food safety and extending product shelf life. Their antimicrobial activity can contribute to the control of foodborne pathogens across different food matrices (228).

In meat and poultry products, postbiotic preparations have been investigated for their antimicrobial effects and their ability to preserve product quality during refrigerated storage (131, 212, 222). Their incorporation into active films and coatings has also been investigated for shelf-life extension in poultry products (212, 222). In seafood, films incorporating postbiotic preparations have been investigated for their ability to limit lipid oxidation, as reflected by lower TBARS values (216). Related active-packaging systems incorporating antimicrobial compounds, such as nisin have, also demonstrated shelf-life extension in seafood products (229).

In the case of fruits and vegetables, postbiotic preparations are being explored as clean-label alternatives to conventional chemical sanitizers. Postbiotic-enriched coatings can delay postharvest deterioration and contribute to maintaining product quality and extending shelf life (183). Evidence from related edible coating systems also indicates that exopolysaccharide-based coatings combined with antifungal lactic acid bacteria can help control fungal spoilage and preserve the postharvest quality of highly perishable produce (230).

Research on dairy products has mainly focused on the application of postbiotic preparations in coatings. Postbiotic coatings have been investigated in cheese production for their potential to improve product quality and preservation (231). Related edible film and coating systems incorporating probiotic or bacteriocin-producing lactic acid bacteria have also demonstrated antimicrobial potential in food applications (199).

Overall, current evidence indicates the potential of whey-derived postbiotic preparations and associated fermentation-derived bioactive compounds as functional components in active food packaging systems, contributing to improved food safety, extended shelf life, and sustainable valorization of dairy by-products (3, 190). Nevertheless, the available evidence remains heterogeneous with respect to the origin and composition of the preparations, packaging matrices, target foods, and the storage conditions. Further studies using well-characterized whey-derived postbiotic preparations under realistic food-storage conditions are needed to establish their efficacy and support their broader application in active food packaging.

### 9.6 Challenges and regulatory considerations

The application of postbiotic preparations in active packaging systems is challenged by the inherent variability of their composition and biological activity, which depends on the microbial strain, fermentation substrate, and processing conditions (51, 143). In whey-based systems, the differences between sweet and acidic whey may further influence the metabolite composition and antimicrobial performance, making standardization an important prerequisite for consistent packaging functionality (3, 27). Although postbiotic preparations are generally more stable than live probiotics, their bioactive constituents may still be affected by processing and storage conditions, which should be considered during their incorporation into active packaging systems (184).

From a regulatory perspective, there is currently no dedicated regulatory framework specifically for postbiotic preparations, and their classification and safety assessment depend on their intended use and applicable regulatory category (174, 192). When incorporated into active food packaging systems, additional requirements applicable to food-contact and active packaging materials must be considered. In the European Union, active food-contact materials are subject to the general requirements of Regulation (EC) No. 1935/2004 and the specific provisions of Commission Regulation (EC) No. 450/2009 on active and intelligent materials and articles intended to come into contact with food (232, 233). In the United States, the regulatory status of substances used in food-contact materials depends on their intended use and may involve an effective Food Contact Substance Notification (FCN), applicable food additive regulations, GRAS status, or other relevant exemptions under the FDA regulatory framework (234). Safety assessment should also consider the potential migration of active substances or their constituents into food and the resulting consumer exposure (233, 234). Regardless of the regulatory pathway, standardized characterization of postbiotic preparations, appropriate safety assessment, and evaluation of migration behavior remain important prerequisites for their successful application in active food packaging (174).

## 10 Conclusion

This review demonstrates that whey valorization through microbial fermentation represents one of the most promising strategies for transforming a dairy by-product with a high environmental burden into a valuable source of fermentation-derived bioactive compounds and, where inactivation and characterization criteria are met, defined into postbiotic preparations. Owing to its favorable nutritional composition, whey provides an excellent substrate for selected lactic acid bacteria and yeasts, enabling the production of a diverse range of fermentation-derived metabolites and whey-derived bioactive compounds, including organic acids, short-chain fatty acids, bioactive peptides, bacteriocins, exopolysaccharides, vitamins, and microbial biomass, as well as structural cell components that may form part of defined postbiotic preparations following appropriate inactivation and characterization. These compounds and preparations collectively contribute to the functional properties of fermented whey, supporting its application in developing value-added food products.

Current evidence indicates that whey-derived postbiotic preparations and fermentation-derived bioactive compounds exhibit multiple biological activities, including antioxidant, antimicrobial, anti-inflammatory, immunomodulatory, and antihypertensive effects, while and contribute to gut health and metabolic homeostasis. Beyond fermented whey beverages, their favorable stability and functional properties make them promising candidates for incorporation into functional foods and active food-packaging systems, where they may improve food preservation and reduce reliance on synthetic additives. Consequently, whey-derived postbiotic preparations and fermentation-derived bioactive compounds represent an important link between dairy by-product valorization, sustainable food production, and the development of next-generation functional ingredients for the food industry.

Nevertheless, several scientific and technological challenges remain before their broader industrial implementation can be achieved. Variability in microbial strains, fermentation conditions, downstream processing, and postbiotic preparation composition continues to limit direct comparisons among studies and complicates product standardization. In addition, although numerous *in vitro* and animal studies have demonstrated encouraging biological activities, well-designed human clinical trials remain limited, and further evidence is required to establish optimal dosage, long-term safety, and health outcomes.

Overall, the available evidence supports the concept that whey should no longer be regarded merely as a dairy by-product but rather as a sustainable biological resource with considerable potential for the production of fermentation-derived bioactive ingredients and, where the relevant criteria are fulfilled, defined postbiotic preparations. Future research integrating precision fermentation, advanced analytical technologies for the characterization of fermentation-derived bioactive compounds and postbiotic preparations, clinical validation, and harmonized regulatory frameworks will be essential to facilitate their commercialization and to fully exploit their potential within sustainable food systems, functional nutrition, and active food packaging applications.
